# Morphological differences in populations of *Jacobaea erucifolia*: Genetic differentiation, phenotypic plasticity or ecotypes?

**DOI:** 10.1371/journal.pone.0332808

**Published:** 2025-09-23

**Authors:** Barbara Gawrońska, Małgorzata Marszałek, Piotr Kosiński, Joanna Zeyland, Leszek Bednorz

**Affiliations:** 1 Department of Biochemistry and Biotechnology, Faculty of Agriculture, Horticulture and Biotechnology, Poznań University of Life Sciences, Poznań, Poland; 2 Department of Botany, Faculty of Agriculture, Horticulture and Biotechnology, Poznań University of Life Sciences, Poznań, Poland; 3 Institute of Dendrology, Polish Academy of Sciences, Kórnik, Poland; Southeastern Louisiana University, UNITED STATES OF AMERICA

## Abstract

Accurate taxonomic classification is essential for effective conservation strategies, especially for rare and endangered species. Traditionally based on morphology, such classifications may be challenged by recent advances in molecular tools that reveal hidden genetic structure or lack thereof. *Jacobaea erucifolia* is a declining species in Poland, primarily threatened by habitat loss, fragmentation, and competition with invasive species. Although two subspecies—*erucifolia* and *tenuifolia*—have been identified in Poland based on leaf morphology and ecological preferences, the taxonomic status of these forms remains unclear. This study aimed to evaluate the genetic variation within ten Polish populations of *J. erucifolia* and assess whether the observed morphological differences correspond to genetic divergence. Based on morphology, four populations were classified as subsp. *erucifolia* and six as subsp. *tenuifolia*. Reference populations of confirmed subspecies *tenuifolia* were included from Slovakia, Hungary, and the Netherlands. Genetic analyses were conducted using amplified fragment length polymorphisms (AFLPs), chloroplast DNA restriction fragments (RFLP), cpDNA, and nuclear internal transcribed spacer (ITS) ribosomal DNA sequences. The results revealed a high level of admixture and no significant genetic differentiation among Polish populations, regardless of their initial morphological classification. All Polish populations formed a single genetic group, distinct from foreign *tenuifolia* samples, although no diagnostic genetic markers were identified to separate these two groups (subspecies) of *J. erucifolia* definitively. Moreover, sequence data showed no differences among all analyzed individuals, further challenging the validity of subspecies differentiation. These findings suggest that the morphological variation observed within Polish populations likely reflects phenotypic plasticity rather than subspecies-level divergence. Consequently, subspecies *tenuifolia* does not appear to occur in Poland, and observed differences between Polish populations and those from neighboring countries may represent ecotypic variation. Our study underscores the necessity of integrating genetic and morphological data when resolving taxonomic ambiguities, especially in the context of conservation planning. Future research involving broader geographic sampling and deeper analysis of hybridization patterns will help clarify the evolutionary history and and population dynamics of *J. erucifolia.*

## Introduction

Accurate taxonomic identification, particularly at the intraspecific level (e.g., subspecies), plays a crucial role in understanding evolutionary processes, such as local adaptation and incipient speciation, and is essential for effective conservation planning. In taxa with wide distributions, morphological variability and hybridization potential, subspecies classifications may reflect underlying ecological divergence, cryptic genetic structure, or phenotypic plasticity. At the same time, anthropogenic changes such as habitat fragmentation, invasive species, and climate shifts exert increasing pressure on natural populations. Then assessing genetic variation within and among populations is essential for predicting their potential to adapt and persist under such changing conditions.

Senecioneae is the largest tribe in the family Asteraceae, represented by over 160 genera (more than 3,000 species), with new genera still being added [1,2]. The tribe is a diverse and taxonomically complex characterized by broad ecological amplitude [3], morphological variation, and frequent hybridization. *Senecio* L. is the largest genus in this tribe and one of the largest genera of flowering plants containing very different species, some are poisonous [4], while others are ornamental or have antimicrobial properties used in folk medicine [5,6]. According to various authors, genus contains 1,250–1,400 species [2,3,7–11], in Poland is represented by 11 species [12] or 27 species [13] depending on the classification in current taxonomy..

The large size of the genus, together with the extraordinary morphological variation both between and within species of *Senecio*, as well as widespread interspecific hybridization in this genus and other genera of Senecioneae [11,14,15], have greatly hampered attempts to create infrageneric classifications of *Senecio*. Furthermore, the considerable diversity of cytotypes within the tribe [2,16,17] contributes to its complex taxonomy and poorly understood evolutionary history. Recently, molecular and taxonomic studies [2,3,7,11,18,19] have shown the dynamic nature of classifications within the tribe Senecioneae with some groups being split off while others have been merged [20]. A notable example is the genus Jacobaea Mill., which now encompasses 45 species, including *J. erucifolia*, formerly classified within *Senecio* sect. *Jacobaea* (Mill.) Dumort [18].

*Jacobaea erucifolia* (L.) G.Gaertn., B.Mey. & Scherb. is a highly variable taxon with a range that extends across a large part of Europe, as well as Western and Central Asia. In Poland, it is mainly found in the southeastern part of the country and generally has a scattered distribution. Moreover, it was, until recently, a poorly recognized species, often even incorrectly identified due to its resemblance to the closely related *Jacobaea vulgaris*. Depending on the source, two subspecies are most commonly distinguished within this species in studies concerning European flora: subsp. *erucifolia* and subsp*. tenuifolia* (J. Presl & K. Presl) [21]. According to Iamonico and Managlia [8], even four subspecies are distinguished. In addition to the two mentioned above, two others: subsp*. arenarius* Soó and subsp. *praealta* are recognized.

Podsiedlik et al. [21,22], based on morphological analyses, demonstrated the presence of both subspecies (*erucifolia* and *tenuifolia*) in Poland. According to the authors, of the 31 inventoried preserved sites, four represent the subspecies *erucifolia* (an Atlantic taxon found in the western part of Poland, in Pomerania), while the remaining 27 represent the subspecies *tenuifolia* (a subcontinental taxon with a scattered distribution in the southern regions of Poland). Similar data, obtained from the morphological variability of the leaves of the studied herbarium materials, were published by Kruk and Sobisz [23]. All of these studies agreed that the vast majority of specimens examined were of the subspecies *tenuifolia*, while specimens designated as *erucifolia* constituted a minority. On the other hand, the presence of these intraspecific taxa in Poland raises some doubts, as it is not included in the available data on *J. erucifolia* localities. According to the only map of the distribution of *J. erucifolia* in Europe [24], only the nominative subspecies *erucifolia* is recorded in Poland. Furthermore, preliminary genetic analyses employing RAPD markers, conducted on material from 10 of the 23 previously morphologically analyzed populations (comprising 100 specimens), did not support the differentiation of the subspecies. RAPD markers showed that populations (considered to be subspecies*. tenuifolia*) clustered together with populations of subspecies. *erucifolia* in two main groups, which appeared to correlate more strongly with habitat characteristics than with taxonomic distinctions (see Supplementary material). While morphological studies identified these subspecies as distinct [21], the genetic evidence did not corroborate such differentiation. It is important to note that this research was limited to material currently available within Poland, without comparative analysis involving typical representatives of these subspecies from their broader geographic ranges. Due to a high variation between and within *Jacobaea* species, morphological discrimination between them is sometimes problematic and often prevents unequivocal identification. Additionally, inter-specific morphological differences may be blurred by the propensity for interspecific hybridization, a common phenomenon within this genus in natural habitats. Based on the results obtained, it was hypothesized that there are no two different subspecies in the Polish flora, and the morphological variability among populations likely reflects environmental adaptation, such as phenotypic plasticity, the development of ecotypes, or hybridization. This interpretation aligns with observations by Walter et al. [25] who documented significant differences in leaf morphology among *Senecio* ecotypes – one of the primary features used to distinguish the putative subspecies of *J. erucifolia* in Poland.

The concepts of species and subspecies are very important for understanding the evolution and ecology of living organisms and are now equally important for ensuring their protection and management [26,27]. Creating a subspecies category is also important for understanding geographic variation and speciation. The taxonomic position of subspecies has been the subject of long-standing disputes, mainly due to frequent inconsistencies between genetic results and subspecies identification based on morphological data [25,26,28–35] which raises additional questions regarding the validity of a number of subspecies and may lead to changes in taxonomy. Until now, the widely accepted definition for subspecies was that of Mayr & Ashlock [30]: *“A subspecies is an aggregate of phenotypically similar populations of a species inhabiting a geographic subdivision of the range of that species and differing taxonomically from other populations of that species.”* However, a kind of molecular revolution has changed the traditional methods (morphological features) of subspecies classification through the use of genetic analyses [26]. Currently, it is assumed that the designation of a subspecies should result from the consistent distribution of many independent genetic characteristics. Thus, subspecies are identified as geographically and genetically distinct populations that exhibit distinct phenotypic differences [27].

Morphological characters for a long time have been practically the main criterion used in plant systematics. However, we must to take in to account that they may be influenced by environmental conditions, and then the morphological differences may be due to differences in habitat rather than being a sign of genetic differences between the groups analyzed [7]. Different species and subspecies, especially those sharing similar habitats, often hybridize with each other, which may lead to phenotypic changes and frequently results in challenges or inaccuracies in taxonomic identification. The occurrence of hybrids with intermediate morphological appearance may lead to the definition of an excessive number of species names within the genus, nomenclature problems and discrepancies in taxonomic approaches [4,19,36]. In such a situation, ambiguities in the species complex can be clarified by molecular investigations [37]. The use of environmentally independent DNA markers can provide more accurate information on genetic diversity, provide insight into speciation processes, and in combination with the analysis of morphological characters is fundamental for taxonomy at the species level.

The most common molecular methods include analyses of organelle genomes (cpDNA and mtDNA), sequence comparison of nuclear ribosomal DNA internal transcribed spacers (ITS) regions, and clustering-based methods that incorporate multilocus genotype data, such as randomly amplified polymorphic DNA (RAPD) [38] or amplified fragment length polymorphism (AFLP) [39]. AFLP, in particular, is a robust and versatile tool for investigating genetic variation. It is widely utilized in genotyping studies, as well as in assessments of population differentiation, and genetic diversity across a variety of organisms, including members of the genus *Senecio* [17]. High levels of polymorphism and high degrees of discrimination are the main advantages of AFLP in distinguishing closely related accessions and assessing genetic diversity within populations. Crucially, the estimation of diversity with this method depends on the number and genomic distribution of markers used and is entirely independent of morphological characteristics. Compared to alternative approaches, AFLP generates a higher number of products (polymorphic fragments and unique bands) in a single reaction and is highly reproducible. All of these methods have already been used to assess relationships, genetic differentiation, between individuals, to demonstrate introgression, and have been successfully applied in hybridization studies within different Senecioneae species [4,11,40–44]. The current study will employ these methods to address the research objectives.

Environmental factors can influence many aspects of plant morphology, and some of the observed differences may arise from various non-genetic causes such as local habitat conditions or introgressive hybridization. Plants exhibit high plasticity in response to environmental stimuli, and many taxa below species rank are described solely based on their morphology. From a taxonomic standpoint, studying such variation is crucial. Moreover, according to Chevin et al. [45], the persistence of species in new or changing environments depends on the ability to adjust phenotypes in response to environmental variability. Importantly, such phenotypic plasticity can generate distinct morphological forms from a single genotype depending on the conditions [46,47]. Understanding phenotypic plasticity is also important for explaining how some taxa—including those with uncertain taxonomic status—may establish in new or disturbed habitats, as is the case for several *Jacobaea* populations observed in altered environments.

Widespread changes in the natural environment, including variations across gradients of latitude, longitude, and altitude, significantly influence the selection of vegetation types and the species found within them [48]. Locally, changes in the plant environment may concern soil type, humidity, or temperature, and influence the adaptation of local types, which adapt to environmental changes through phenotypic plasticity or adaptive genetic divergence. According to Mayr [49], adaptation along an environmental gradient, with or without gene flow, may be the first step leading to the emergence of fully reproductively isolated forms, known as biological species.

Given the need to understand how phenotypic traits respond to environmental variation, especially in species showing morphological divergence without clear genetic structure, it becomes important to investigate how genotypes relate to phenotypes across different habitats, as well as the processes influencing such patterns [25]. Equally important is understanding the interactions between phenotypic plasticity and natural selection, and how and when populations specialize in particular environments. The genus *Senecio* is an ideal model, with species that naturally interbreed, inhabit different environments, and undergo phenotypic differentiation and adaptation [25]. Within the genus, there are several examples of radiation mechanisms. Some of these have been described as introgressive hybridization [50], local adaptation [51], or hybrid speciation [52,53]. For example, introgressed lowland species, such as *S. chrysanthemifolius* and *S. ovatus*, have been observed to move to higher altitudes, possibly in response to recent global warming, displacing their higher-altitude counterparts from these areas [48].

Genetic variation is essential for populations to persist in the face of environmental pressures, including those driven by human activity. Habitat fragmentation, agricultural intensification, and the introduction of invasive species can reduce effective population size and gene flow, leading to a loss of genetic diversity. This, in turn, limits a population’s ability to adapt to new or changing environmental conditions. In this context, evaluating the genetic structure of populations is crucial for understanding both their evolutionary potential and their vulnerability.

In the face of global climate change and human activity, the significant role of hybridization cannot be overlooked when considering adaptation and the occupation of new habitats. On one hand, hybridization can lead to the formation of new species and adaptive traits (such as the ability to occupy new niches and expand range) [54,55]. On the other hand, it may also lead to the replacement of native species by invasive ones [56,57], potentially resulting in a loss of biodiversity. In the absence of reproductive isolation, hybridization can reduce or eliminate differentiation (introgressive hybridization; [58], leading to the genetic erosion of parental gene pools [59]. In the case of local endemic species and small isolated populations, introgressive hybridization can pose a significant threat. Human activity also plays a significant role in promoting hybridization [60], which, driven by anthropogenic factors, is one of the key problems for conservation. In light of this, maintaining existing biodiversity has become a major challenge [60–62]. Moreover, environmental changes and the introduction of exotic species can lead to demographic or genetic swamping, threatening the integrity of small populations or rare species [60,63]. Due to increasing global changes, the rate of hybridization appears to be rising [62,64].

Available data from literature, herbarium materials, and field inspections have shown that the process of *Jacobaea*. *erucifolia*’s (hoary ragword) decline is progressing, largely due to the intensification of agriculture and the elimination of fallow lands, which are currently the most frequently recorded habitat of this species. Habitat loss is further exacerbated by the spread of invasive species, such as *Solidago gigantea* and *Calamagrostis epigejos*, which have been extensively observed in areas previously occupied by *J. erucifolia*. as well as the effects of urbanization in areas where the species once occurred in Poland [22]. The species is now rare at the national level and deserves special protection [22,23].

In this study, we employed a combination of molecular tools— AFLP fingerprinting, cpDNA restriction site variation, and sequence analysis to investigate the genetic status of Polish populations of *J. erucifolia* in the context of their variability within the species’ European range.. We aimed to test whether the morphological variation observed in these populations corresponds to genetically distinct subspecies or instead reflects adaptation to environmental conditions or introgressive hybridization. This approach reflects broader questions in plant taxonomy and conservation regarding the reliability of morphological criteria for subspecies recognition under anthropogenic pressure. Our findings also contribute to conservation planning by clarifying the genetic distinctiveness of declining Polish populations. Specifically, the objectives of this study were: (1) to assess the level of genetic variation in Polish populations of *J. erucifolia* in comparison with populations from other countries within the continuous distribution range of the species, and (2) to re-evaluate the taxonomic validity of the subspecies *tenuifolia* in Poland, using comparative material. from Slovakia Hungary and the Netherlands.

## Materials and methods

### Plant material

A total of 127 individuals of *J. erucifolia* (representing both putative subspecies) were included in this study, originating from 15 populations in total (Table 1). The sampling covered ten populations from Poland, four from Hungary, and one from the Netherlands. Among the Polish populations, four represented *J. erucifolia* subsp. *erucifolia* (Western Pomerania) and six were described as *J. erucifolia* subsp. *tenuifolia*. Each Polish population was represented by ten individuals, selected to ensure wide geographic and ecological coverage, based on morphological classification and site accessibility. The Hungarian and Dutch populations have not been formally assigned to subspecies; however, based on morphology, they most likely correspond to subsp. *tenuifolia* (tentatively referred to as *J. erucifolia* in Table 1). From each of the 15 populations, ten individuals were sampled (although in a few cases only three or five individuals were available). All of these samples were collected during fieldwork. Additionally, six individuals of *J. erucifolia* subsp. *tenuifolia* originating from six different Slovakian regions were included, based on herbarium material deposited in the Herbarium NI, Department of Botany, Faculty of Agrobiology and Food Resources, Slovak University of Agriculture, Nitra, Slovakia.

**Table 1:** List, origin and number of individuals in the populations studied.

Locality	Acronym	Latitude N	Longitude E	Number of specimens	Habitat
***J. erucifolia* subsp. *erucifolia***					
Poland, Kłosów	K	52.733	14.450	10	fallow with *Calamagrostis epigejos*
Poland, Moczyły	M	53.317	14.467	10	plant community with *Calamagrostis epigejos*
Poland, Szczecin Skolwin	S	53.600	14.600	10	meadow with single shrubs
Poland, Szczecin Stołczyn	St	53.500	14.600	10	nitrophylic herb vegetation
***J. erucifolia***					
Hungary, Tápiószentmárton	HT	47.364	19.808	5	secondary mesotrophic grasslands
Hungary, Ivan	HI	47.432	16.941	3	secondary mesotrophic grasslands
Hungary, Tolna	HL	47.393	18.744	5	secondary mesotrophic grasslands
Hungary, Tápiószele	HP	47.335	19.844	5	secondary mesotrophic grasslands
Netherlands, Zetten	NZ	51.938	5.693	3	road verge in river valley, clay
***J. erucifolia* subsp. *tenuifolia***					
Poland, Brwinów	B	52.133	20.700	10	nitrophylic herb vegetation
Poland, Brzeźno	Brz	51.150	23.600	10	abandoned garden, set-aside
Poland, Pęczelice	P	50.433	20.783	10	fallow field
Poland, Parchatka	Pa	51.350	22.000	10	plant community with *Solidago gigantea*
Poland, Sielec	Si	50.350	20.067	10	fallow field
Poland, Sitno	Sn	51.367	16.367	10	plant community with *Calamagrostis epigejos*
Slovakia, Salka	SS	47.888	18.719	1	edge of vinevards, loess
Slovakia, Búč	SB	47.805	18.511	1	loess
Slovakia, Šurany	SU	48.087	18.126	1	saline pastures
Slovakia, Leles	SL	48.483	22.035	1	pastures, sub-saline soil
Slovakia, Večelkov	SV	48.160	19.918	1	abandoned pastures
Slovakia, Zlatná	SZ	47.767	17.938	1	abandoned saline pastures
***J. vulgaris***					
Poland, Golęcin	G	16.850	52.417	10	secondary mesotrophic grasslands
Poland, Puławy	Pu	21.967	51.400	9	secondary mesotrophic grasslands
Netherlands, Plasmolen	NP	51.739	5.921	10	mesotrophic meadow/pasture

*Note*: the Hungarian and one Dutch populations were not initially defined in terms of subspecies and are provisionally described in table as *J. erucifolia*. The order of populations in the table follows the preliminary morphological classification available at the time of sampling. For some Polish populations, the subspecies assignment also remains tentative.

Due to the small number of individuals (single specimens) from particular locations, the samples from Slovakia were not treated as populations but as a reference group (in most statistical analyses the required number of individuals is at least three or more). Twenty-nine individuals of *J. vulgaris* (2 populations from Poland and one from the Netherlands) served as an out-group. In our abbreviation system, the first letter denotes the country of origin for foreign populations, while the subsequent letters refer to a specific locality, typically a nearby town (both capital letters). Polish populations are labeled directly by sampling site names. The same material has been previously used in morphological analyses as well as in our earlier study on hybrids between *Jacobaea erucifolia* and *J. vulgaris*. In the current study, we intentionally did not use the letter “P” to denote Polish populations in order to maintain consistency in the naming and localization system applied to all samples. Details of the specimens tested are provided in Table 1.

The choice of *J. vulgaris* as an out-group was related to the results of previous studies documenting the occurrence of spontaneous hybridization, between *J. vulgaris* and *J. erucifolia*, especially in areas where both species occurred. Moreover, our analyses showed that some individuals in populations previously described as pure were probably also of hybrid origin, backcrosses to *J. erucifolia* and *J. vulgaris* [65]. The fact that both species are genetically different and frequently interbreed facilitates the assessment of the studied *J. erucifolia* populations in terms of introgression.

### DNA extraction

Young leaves collected in the field were dried with silica gel. The total genomic DNA of all 156 specimens was extracted following the method described by Doyle and Doyle [66]. The contaminating RNA was removed by digestion with RNase A. DNA quality and concentration were estimated by electrophoresis and spectrophotometry. The extracted DNA was adjusted to a standard concentration of 20 ng/μl, and used as a template in polymerase chain reaction (PCR).

### AFLP procedure

In the present study, AFLP analysis was performed to estimate the level of variability and assess the genetic relationships among studied populations collected from different localities. All 156 individuals, morphologically identified as belonging to either of the two subspecies of *J. erucifolia* or *J. vulgaris* were included in the AFLP analysis following the procedure described by Vos et al. [39] with some modifications. Individuals of *J. vulgaris* served as the outgroup. Details of preparation and program of PCR reactions were described by Gawrońska et al. [67].

After screening of 15 selective primer pair combinations, four combinations *Eco*RI/*Mse*I (+3/ + 3) were selected that were easy to score and gave the high polymorphism and reproducibility of AFLP profiles. Primer pairs: (E-ACA/M-CAG, E-ACA/M-CAT, E-ACA/M-CCG, and E-AGG/M-CTT) were further screened over all individuals. Each reaction was repeated at least two times to test the reproducibility of AFLP profiles. Selective amplifications were performed using the primer EcoRI labeled with the fluorescent dye 6-FAM. Products of selective amplification were separated on POP7 on an ABI Prism 3500 capillary sequencer. GeneScan 600 LIZ-labeled size standard (Applied Biosystems) was used for fragment sizing. Fluorescent AFLP patterns were evaluated using GeneMarker software version 1.6 (Softgenetics LCC) using default parameters according to the manufacturer’s recommendations as optimal for AFLP markers. Fragments, in the range between 50–500 bp, were automatically scored as present (1) or absent (0). However, all electrophoretograms were manually corrected after visual inspection to eliminate possible misinterpretations of the automated routine. Only fragments that could be unambiguously scored were recorded and used for the generation of binary data matrix.

### Data analysis

Genetic diversity within the analyzed populations was characterized using various parameters. The total number of AFLP multilocus genotypes, distribution of bands across populations as well as the average number of bands per primer were examined. Genetic divergence among analyzed genetic groups was also estimated by calculating the percentage of polymorphic markers (bands present in at least one but not all specimens), the number of monomorphic bands (present in all specimens under the study), bands shared among subspecies but also group of unique bands which were present in at least one individual in a given taxon and not present in any other [68]. However, in the case of analyses confirming species affiliation (here molecular distinctiveness of the *tenuifolia* subspecies), it is of primary importance to find diagnostic markers that are a subset of all unique markers but restricted to a certain taxon and present in all of its individuals.

The binary matrix of AFLP phenotypes was the basis for calculating Dice similarity. Then genetic similarity was transformed into genetic distance using the formula *Dij *= 1–*Sij*. The distance matrix was used subsequently for clustering such as construction of the unrooted neighbour-joining (NJ) dendrogram and phylogenetic network based on the Neighbor-Net algorithm using SplitsTree4 v.4.13.1 software [69]. Compared to other methods of clustering, Neighbor-Net [70] better visualizes relationships among individuals. The above analyses were supplemented by the Unweighted Pair Group Method with Arithmetic Mean (UPGMA) dendrogram and a principal coordinate analysis (PCoA), both performed in PAST 3.15 [71]. The bootstrapping method was used to calculate a support value for each node on the dendrograms (1000 replicates).

Finally, to quantify the distribution of genetic variation among and within populations of *J. erucifolia* the hierarchical analysis of molecular variance (AMOVA; [72]), was performed. Analyses were carried out in GenAlEx 6.1 [73]. The isolation-by-distance (IBD) analysis was carried out in GenAlEx by testing the correlation between the matrices of genetic distance (PhiPT values) and the pairwise geographical distance using the Mantel test. The significance of IBD was assessed through 1,000 permutations.

The genetic structure of species (identification of genetically homogeneous groups) was analyzed using a Bayesian model-based approach implemented in STRUCTURE, version 2.3.4 [74]. STRUCTURE analysis was performed based on the admixture model, with the recessive allele option set to 1 as AFLPs are the dominant markers [75]. Ten independent repetitions were performed for each number of groups (K) ranging from one to five, with 105 steps burn-in followed by 2 × 105 MCMC iterations. The consistency of the results in 10 replicates of analyses was assessed using CLUMPAK [76], and the best number of clusters was determined by the Evanno ΔK method [77] implemented in the online STRUCTURE HARVESTER program [78].

The results of the analyses described above are visually represented using a consistent color-coding system: red for *J. vulgaris*, blue for *J. erucifolia* subsp. *erucifolia*, green for *J. erucifolia* subsp. *tenuifolia*, and sea blue for individuals/populations not assigned to a subspecies during collection (but most likely representing *J. erucifolia* subsp. *tenuifolia* based on photographic documentation).

### Chloroplast DNA haplotypes

The usefulness of universal primers for assessing chloroplast polymorphism among plant populations was demonstrated for many species. These universal primers are located in coding regions of cpDNA, separated by more variable intergenic regions [79,80]. One of the popular methods useful in population analyses is the PCR-RFLP method (combining both the PCR and RFLP techniques), which allows for the distinction of genotypes based on the presence or absence of restriction sites in the amplified DNA.

The total DNA of individual plants was used in PCR reaction, the variation of the chloroplast genome was tested using a set of universal primers. In a first step, four pairs (LF, CS, HK, and K1K2) of chloroplast primers were used to identify and screen polymorphism on individuals from the populations investigated. Three of the pairs of primers mentioned above were selected because of their good amplification pattern. They were included the non-coding region between *trn*L and *trn*F genes of cpDNA, CS region *psb*C and *trn*S and HK region *trn*H *trn*K. The first fragment was amplified using universal primers described in Taberlet et al. [81], while the CS and HK regions were amplified with pairs of universal primers described in Demesure et al. [79]. (Table 2)

**Table 2:** Chloroplast DNA (cpDNA) universal primer pairs used in this study.

Primer 1	Primer 2	Abbreviation	Ref.
**trnK [tRNA-Lys(UUU) exon 1**	**trnK[tRNA-Lys (UUU) exon 2**	**K1K2**	**1**
**trnH [tRNA-His (GUG)]**	**trnK [tRNA-Lys (UUU) exon 1**	**HK**	**1**
**psbC [PSII 44 KD protein]**	**trnS [tRNA-Ser (UGA)]**	**CS**	**1**
**trnL [tRNA-Thr (UAA)]**	**tmF [tRNA-Phe (GAA)]**	**FL**	**2**

^1^ Demesure et al. [79]; 2 Taberlet et al. [81]. The pairs of primers used for all populations are in bold.

PCR reactions were carried out in a final volume of 25 μL containing 20 pmol each of primer and about 40 ng of template DNA using the KAPA3G Plant PCR Kit (Sigma Aldrich). Reactions were performed in a PTC-100 thermal cycler (MJ Research), according to the previously described program [65]. Annealing temperature and the extension time were dependent upon the primers used and the length of the PCR product. The efficiency and quality of amplification were verified by electrophoretic separation of PCR products in 1.5% agarose gel. The size of the amplified fragments was estimated using a 100 bp ladder DNA marker (Thermo Fisher Scientific). PCR products (5µl) obtained for several samples representing both putative subspecies of *J. erucifolia* were digested with 18 restriction endonucleases: *Alu*I, *Hinf*I, *Rsa*I, *Tru*I(*Mse*I), *EcoR*I, *Bsu*RI, *Hha*I, *Hind*III, *Hpa*II, *Tag*I, *Sau*3a, *Mbo*I, *Eco*RV, *Kpn*I, *Sac*I, *Pst*I, *Dra*I and *Alw*26I under conditions recommended by the enzyme supplier (Thermo Fisher Scientific). The enzymes used for digestion were selected randomly. Based on their ability to cut and reveal polymorphism in cpDNA regions, four chloroplast fragment-enzyme combinations (CS–*Alw*26I, CS–*Bsu*I, HK–*Rsa*I, and HK–*Hinf*I) that exhibited readable polymorphism were selected. In a second step, only these combinations were used to characterize a total of 127 individuals from 15 populations and six individuals representing subspecies *tenuifolia* from Slovakia. Restriction digests were separated by electrophoresis in 8% polyacrylamide gels using Tris-borate EDTA buffer (0.5) at 70 V overnight and visualized by silver staining. 100-bp ladder marker (Thermo Fisher Scientific) was used as a size marker on each gel To determine relative size differences, after the first size measurement, the different variants for each polymorphic locus were re-ran on the same gel. cpDNA variants at each locus were numbered, the numbers increase from the highest to the lowest molecular weight fragments. A binary matrix was created based on the presence or absence of each restriction fragment at each polymorphic site. The similarity matrix (estimated using the simple matching coefficient from the binary matrix) was used to construct a dendrogram by unweighted pair group method average (UPGMA). Variation in the restriction patterns was interpreted as a haplotype and the haplotype nomenclature was used as described by Petit et al. [82].

### Sequence analysis of chloroplast DNA

In cases where the results raise doubts, particularly regarding the taxonomic position of certain samples or the possibility of introgression, the amplified fragments of an intergenic spacer between the *trn*L (UAA) exon 3’ and *trn*F (GAA) were purified using a DNA cleanup kit (Clean-Up, A&A Biotechnology) and sequenced in both directions The obtained sequences were compared to each other and with those previously obtained [65]. All DNA sequences were visualized and analyzed using SnapGene Viewer 6.0.2. (https://www.snapgene.com/snapgene-viewer), visually adjusted if necessary and automatically aligned using the program MEGA 11 [83]. The new sequences generated in this study have been deposited in GenBank (accession numbers: PQ509766- PQ509769).

### ITS sequence analysis

The entire ITS region (ITS1, 5.8S, ITS2) was amplified using the universal ITS1 and ITS4 primers developed by White et al. [84]. Reaction conditions were described previously by Gawrońska et al. [67]. Both the efficiency and quality of amplification were verified by the electrophoretic separation of 5 µL of PCR products in 1.5% agarose gel. After purification as described above, PCR products were sequenced in both directions using the same primers as during amplification. The aim of the analysis was to compare sequences obtained for reference individuals morphologically defined as *tenuifolia* subspecies from Slovakia, with those previously obtained (accession numbers: OP764514–OP764528) for *erucifolia* species (both putative subspecies in Poland; [65]) and to detect possible differences.

## Results

### AFLP analyses

To evaluate patterns of genetic diversity and structure among populations, we first analyzed AFLP data, which provides multilocus genome-wide information independent of sequence assumptions. The study included 156 samples from 18 populations (including three populations of *J. vulgaris*) from Poland and several European countries. Fifteen of them were represented by both putative subspecies of *J. erucifolia*. Six individuals from Slovakia, referred to as subspecies *tenuifolia* were used as the reference group.

Among 15 AFLP primer combinations prescreened, four were selected. AFLP fingerprinting using the 4 selective primer combinations yielded 687 clearly resolved and unambiguously scored fragments including those characteristic for populations of *J. vulgaris* consisting an out-group (54 products). In populations of *J. erucifolia* (both subspecies), 625 out 633 (98%) were polymorphic across all populations (127 accessions). The number of polymorphic bands per primer combination ranged from 149 to 172, with an average of 158 bands. The percentage of polymorphic bands varied among the different primer combinations. The highest percentage of AFLP polymorphisms was obtained for *Eco*ACA/*Mse*CAT and *Eco*ACA/*Mse*CCG combinations, where 149 out of 151 and 163 out of 169 (98.7 and 96.4%), respectively, bands were polymorphic. The size of the AFLP amplified fragments ranged from 50 bp to 500 bp. In general, the number of polymorphic AFLP fragments characterizing intra-populational variation in Polish populations of *J. erucifolia* regardless of the subspecies was higher than in other populations and averaged 92,5%. The lowest values: 290 polymorphic fragments and a polymorphism of 73% were observed for the NZ population from the Netherlands (Table 3).

**Table 3:** Genetic diversity estimates within the *J. erucifolia* populations (both subspecies) based on 633 AFLP fragments.

Population code	*N*	*N*_poly_	% _poly_
***J. erucifolia* subsp. *erucifolia***			
M	529	517	97.7
S	529	523	98.9
K	543	535	98.5
St	518	502	96.9
***J. erucifolia***			
HT	469	393	83.8
HI	437	343	78.5
HL	488	423	86.7
HP	547	459	83.9
NZ	397	290	73.0
***J. erucifolia* subsp. *tenuifolia***			
P	374	322	86.1
B	458	435	95.0
Sn	527	512	97.2
Brz	512	506	98.8
Si	499	476	95.4
Pa	514	501	97.5
SK (SS, SB, SU, SL, SV, SZ)	455	395	86.8

Names of populations (codes) according to Tab.1. *N-* number of AFLP fragments; *N*_poly_ – number of polymorphic AFLP fragments; %_poly_ – proportion of polymorphic fragments;

*Note*: the Hungarian and one Dutch populations were not initially defined in terms of subspecies and are provisionally described in table as *J. erucifolia*. Individual specimens from Slovakia are treated as a reference group (SK).

In order to confirm the morphological differences between subspecies, the genotypic profiles of these taxa were also examined and checked whether there were any differences in DNA fragments between them. The search for polymorphic private AFLP fragments per population as well as per taxon was carried out. In the *Eco*ACA/*Mse*C*A*G combination, one fragment was noted that met the criteria for defining it as private for the P population, the 399 bp fragment was present in all 10 individuals and absent in the other populations. The presence of a similar fragment 396 bp in *Eco*ACA/*Mse*CCG combination, was also demonstrated for the S population (7 out of 10 specimens). Profile analysis revealed a number of unique products usually, occurring in single individuals only in one or several populations in the group including the *erucifolia* subspecies. In the S population, 14 of them were noted (some were shared with other populations described as *erucifolia* subspecies). A total of 59 unique products were found in the Polish population profiles. However, such fragments also frequently appeared in the Slovak reference group. In comparison with Polish populations, only 11 unique fragments were recorded in the profiles of populations recognized as subspecies *tenuifolia*. As with the previous group, fragments most often occurred in single individuals or in single populations. According to the commonly accepted definition, diagnostic markers were considered to be those that are present in more than 95% of individuals of one taxon and absent in another taxon. None of the mentioned unique fragments met these requirements and could not be classified as a species-specific diagnostic marker.

**Fig 1:**
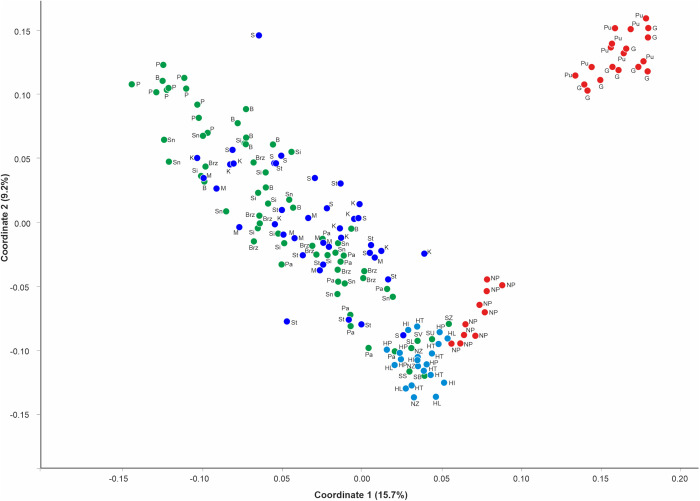
Principal coordinates analysis (PCoA) plot based on the individual genetic distance calculated with 687 AFLP markers.

Cluster analyses were performed based on data from polymorphic AFLP bands including specimens from the out-group. The estimated similarity coefficient based on the combination of 687 markers. The pairwise Dice’s similarity coefficient values between all possible pairs of genotypes ranged from 0.172 for P3 and Pu3 to 0.805 between two *J. vulgaris* accessions. The principal coordinate analysis (PCoA) divided all accessions into three groups between the two first coordinates, which accounted for 24.9% of the total genetic variation (15.7% and 9.2% for the first and second axes, respectively) (Fig 1). The first well-separated group was formed by two Polish populations of *J. vulgaris* (Pu and G served as an out-group), the largest group encompassed individuals of *J. erucifolia* identified on the basis of morphological features as both subspecies in Poland. Most of them formed a loose grouping without a clear division into subspecies or population. The third group, located between them included accessions represented by *J. erucifolia* from Hungary and the Netherlands as well as 6 individuals from Slovakia representing *J. erucifolia* subsp. *tenuifolia* and *J. vulgaris* individuals from the Netherlands. Nevertheless the latter (specimens NP1-NP10) formed a subgroup loosely connected to this group.

The first two axes explain 15.7% and 9.2% of the variation, respectively. Individuals are represented by dots in colors, (red corresponds to *J. vulgaris*, blue to *J. erucifolia* subsp. *erucifolia*, green to *J. erucifolia* subsp. *tenuifolia* and sea blue for individuals/populations not assigned to a subspecies during collection, probably subsp. *tenuifolia*).

**Fig 2:**
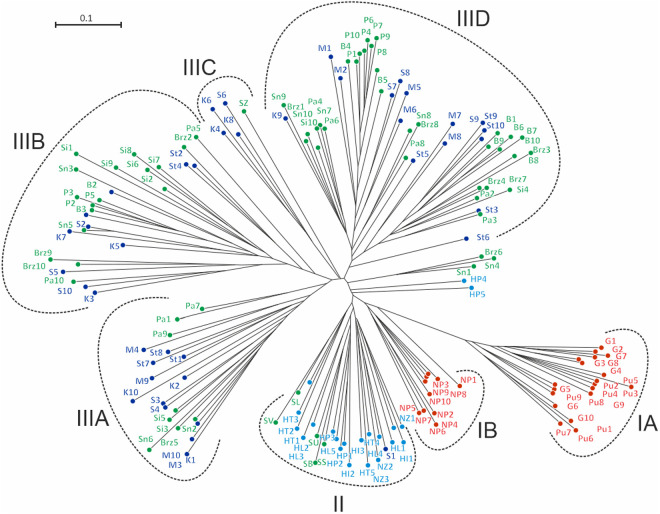
Neighbor-Net derived from AFLP binary matrices of 156 individuals representing *Jacobaea erucifolia* (both subspecies) and *J. vulgaris* (out-group).

Both the UPGMA dendrogram and the Dice Bio NJ network (Fig 2) showed good agreement and confirmed the results of the principal coordinate analysis indicating no separation between *J. erucifolia* subspecies in Poland. Compared to PCoA, although the major clustering pattern was found to be similar, changes in minor grouping were observed. NJ tree revealed three clusters corresponding to the main groups in PCoA. (Fig 1). The Polish individuals of *J. vulgaris* formed a homogeneous group I. As before, individuals from all foreign populations (HT, HI, HL, HP, NZ) and most individuals constituting the reference group (SS, SB, SU, SL, and SV) from Slovakia grouped together and formed group II. The subclaster consisting of individuals of *J. vulgaris* from the Netherlands, previously located within this group, now separated clearly and formed a branch within the *J*. *vulgaris* group, (Polish representatives of this species). All remaining individuals clustered together within the largest cluster III, further subdivided into several smaller subclusters (A, B, C, D). Three of them were heterogeneous with accessions scattered randomly, independently from their taxonomic position and apart from the P (Pęczelice) and B (Brwinów) populations also of their geographic origin. Interestingly, the smallest subclaster IIIC was represented by 3 individuals from Kłosów (K), one from Szczecin Skolwin (S) populations corresponding to the *erucifolia* subspecies, previously described as probably backcrosses [65] and SZ individual from Slovakia representing *tenuifolia* subspecies. Also noteworthy were five individuals not assigned to any group, which were located between the large group IIID and the branches formed by *J. vulgaris*. Three of them (Sn1, Sn4, Brz6) formed a small group, while the remaining two (HP4 and HP5) occupied separate positions without forming one group with them (Fig 2).

Individuals are represented by dots in colors, (red corresponds to *J. vulgaris*, blue to *J. erucifolia* subsp. *erucifolia*, green to *J. erucifolia* subsp. *tenuifolia* and sea blue for individuals/populations not assigned to a subspecies during collection, probably subsp. *tenuifolia*). It should be noted that the Dutch individuals morphologically representing *J. vulgaris* form a separate group, a cluster which, although related to the branch of this species from Poland, is located closer to the cluster formed by the *J. erucifolia* subsp. *tenuifolia*. Accessions codes as in Table 1.

**Fig 3:**
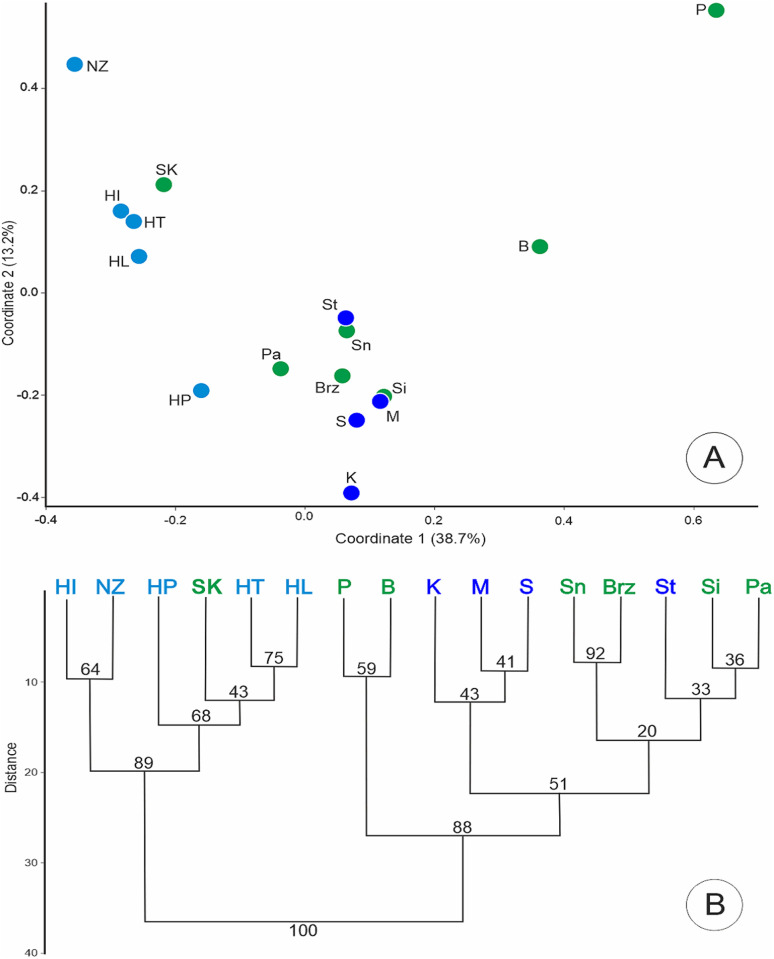
Grouping of populations of *J. erucifolia* subspecies into clusters based on Dice distances calculated from AFLP binary matrices. (A) Scatter plot of PCoA of studied populations. Two first coordinates explain 52.4% of the total observed variation. (B) An unrooted neighbour-joining (NJ) tree, numbers at the nodes indicate bootstrap support. Names of populations refer to Table 1 (SK-code for reference group). Individuals are represented by dots in colors, (blue to *J. erucifolia* subsp. *erucifolia*, green to *J. erucifolia* subsp. *tenuifolia* and sea blue for individuals/populations not assigned to a subspecies during collection, probably subsp. *tenuifolia*).

A similar pattern of genetic diversity was observed in the dendrogram and PCoA based on pairwise genetic distances among populations when the analysis was performed at the population level. The NJ tree revealed two well-supported clusters (bootstrap value of 100%). One group included Polish populations (both putative subspecies), while the second group consisted of populations from Hungary, the Netherlands, and Slovakia (subsp. *tenuifolia*). In both analyses, the position of two populations, P and B, is noteworthy, as they clearly separate from the others (Fig 3). The population from Pęczelice (P) was characterized by significantly lower similarity coefficient values compared to the other populations. The lowest similarity was recorded in relation to the Hungarian HI population (0.666). The remaining populations showed relatively high similarity coefficient values, with a maximum of 0.879 between the Pa and Sn populations.

The analysis of molecular variance (AMOVA) of *J. erucifolia* populations was performed twice, using geographic origin and taxonomic position (subspecies) as the grouping criteria. In both cases, AMOVA attributed most of the genetic variation to within-population variation (83–84%), rather than between populations or subspecies, regardless of the type of analysis (Table 4).

**Table 4:** Analysis of molecular variance (AMOVA) for *J. erucifolia* populations based on AFLP markers.

Source of variance	df	SS	MS	Variance component	% of total variance
Groups based on geographical origin					
Variance among populations	12	3515.137	292.928	19.850	16
Variance within populations	114	11477.856	100.683	100.683	84
Total	126	14992.992		120.533	100
Groups based on taxonomic position					
Variance among subspecies	1	434.907	434.907	2.060	2
Variance among populations	11	3080.229	280.021	18.726	15
Variance within populations	114	11477.856	100.683	100.683	83
Total	126	14992.992		121.469	100

Abbreviations: df – degrees of freedom; SS – Sum of square; MS – mean squares.

**Fig 4:**
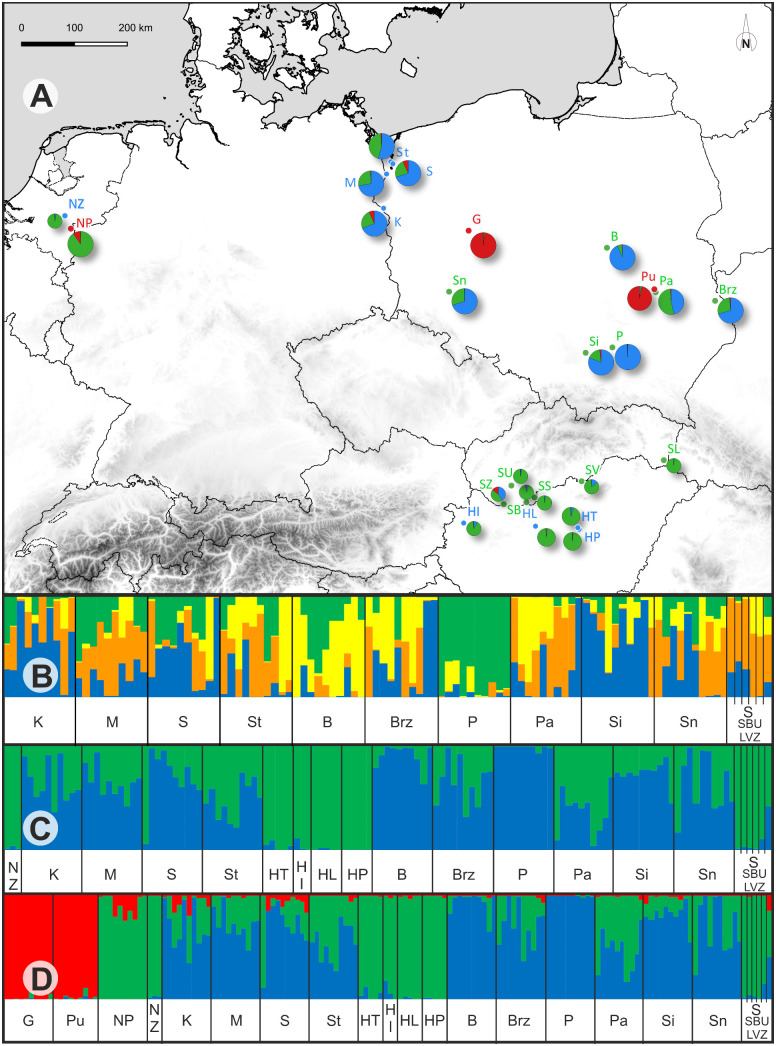
Clustering analysis of AFLP data for *J. erucifolia* (both subspecies) and *J. vulgaris* populations, and their distribution in Poland and other countries. (A) A map with pie charts showing the proportion of membership in each cluster, corresponding to the bottom barplot with STRUCTURE results for K = 3. (B) Clustering analysis using Bayesian analysis (STRUCTURE) for Polish populations and the reference group from Slovakia, for K = 4. (C) Analysis after adding the remaining populations representing subsp. *tenuifolia* from Hungary and the Netherlands, for K = 2. (D) Clustering analysis including all *J. erucifolia* together with populations of *J. vulgaris*, for K = 3. Individuals are represented by columns, with colors representing the proportion of their genome assigned to the inferred clusters in the model-based admixture analysis. Symbols identify the different populations investigated. Additional details for each population are given in Table 1. Map created in QGIS [QGIS Development Team, 2025); land, sea, and administrative boundary layers were sourced from Natural Earth (naturalearthdata.com).

Prior to interpreting the STRUCTURE results, the most likely number of clusters (K) (S2 Fig) for each dataset was estimated using the ΔK method of Evanno [77]. This approach provides a more robust justification than raw likelihood values, which are associated with large variance among replicates. In the STRUCTURE analysis (Fig 4A), which included all Polish populations and individuals from Slovakia, the best-supported number of genetic groups was found to be four (K = 4). Although the studied populations differed in their allele composition, the occurrence of similarly colored segments indicated the presence of shared alleles. The proportion of these shared alleles varied across populations, but did not allow for the identification of distinct groups. However, two exceptions were noted: the nearly homogeneous population P, and two populations (B and Brz), which exhibited an exceptionally high level of admixture (Fig 4B). This analysis step, restricted to Polish and Slovakian populations, provided a direct comparison of Polish material with the only available reference samples of *J. erucifolia* subsp. *tenuifolia*.

**Fig 5:**
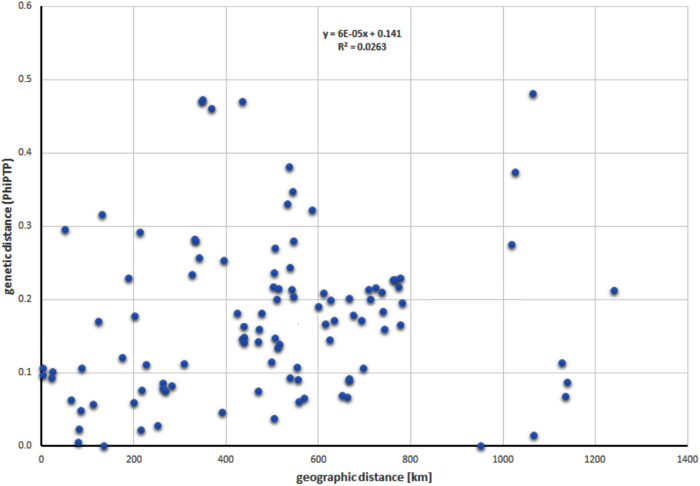
Scatterplot showing the results of correlation (Mantel test) between *Φ*_PT_ coefficient values and geographic distance for *J. erucifolia* (both subspecies) populations.

When the remaining populations from Hungary and the Netherlands were added, the best-supported number of clusters decreased to K = 2. The observed pattern of differentiation corresponded to the division of the samples into two groups (Fig 4C). The first group, encompassing all Hungarian populations, the population from the Netherlands, and individuals from Slovakia (morphologically defined as *J. erucifolia* subsp. *tenuifolia*), was relatively homogeneous. In contrast to most Polish populations, these populations showed minimal variation. The second group included Polish populations represented by both subspecies (defined on the basis of morphology), and showed a clear overlap of the gene pools of both groups, except for populations P and B which exhibited very slight admixture. Based on the Mantel test, no significant correlation was found between genetic (PhiPTP) and geographic distances (R^2^ = 0.026, P = 0.132) (Fig 5).

Given the previously demonstrated spontaneous hybridization and evidence of introgression between *J. erucifolia* and *J. vulgaris* [65], STRUCTURE analysis was also performed after including different *J. vulgaris* populations. When these populations were added, Bayesian clustering revealed that the best-supported number of clusters was K = 3, dividing the samples into three main groups (Fig 4D). Although the results for two of the groups (*J. erucifolia*) were similar to those found for K = 2, slight admixture of *J. vulgaris* was observed in most of the populations. In the third clade, encompassing *J. vulgaris* populations (G, Pu, and Nz from Poland and the Netherlands, respectively), an overlap of *J. erucifolia* gene pools was also observed. Moreover, in the latter population (assigned to *J. vulgaris*), the cluster typical of *J. erucifolia* dominated clearly. The genetic affinity revealed by the STRUCTURE analysis was consistent with the results of the NJ network analysis. All analyses conducted using AFLP markers clearly indicated that the populations from Hungary and the Netherlands are very similar to the representatives from Slovakia, which are classified as the *tenuifolia* subspecies, and were treated as such in subsequent analyses. Some of the Polish populations, which were also assigned to this subspecies based on morphological features, consistently formed a separate group in each analysis, alongside the populations defined as the *erucifolia* subspecies. Furthermore, as these populations exhibited no significant differences, they were collectively referred to as Polish populations of *J. erucifolia* in the subsequent sections.

### Analysis of chloroplast haplotypes

To complement the genome-wide data, we next assessed plastid diversity through cpDNA RFLP analyses, which reveal maternal lineage patterns and are useful for inferring phylogeographic structure. Initially, four universal primer pairs of (LF, CS, HK, and K1K2) were used for cpDNA amplification. However, due to their high amplification efficiency, three primer pairs (*trn*C/*trn*S, *trn*H/*trn*K, and *trn*L/*trn*F) were selected for this study (Table 2). The estimated sizes of the fragments amplified with the CS, HK, and LF primer pairs were approximately 1600, 1800, and 960 bp, respectively. No length polymorphisms were detected in the PCR products using any of the primer pairs tested.

PCR products were digested by six selected restriction enzymes (*Alw*26I, *Bsu*I, *Rsa*I, *Hpa*I, *Thru*I(*Mse*I), and *Hinf*I) to identify polymorphisms, as described by Dumolin-Lapegue et al. [80]. Of the fragment-enzyme combinations, four (CS–*Alw*26I, CS–*Bsu*I, HK–*Rsa*I, and HK–*Hinf*I) yielded clear polymorphic patterns. The LF region, while initially amplified, showed minimal variation among individuals when digested with four enzymes (LF–*Hpa*I, LF–*Rsa*I, LF– *Thru*I(*Mse*I) and LF–*Alw*26I). Therefore RFLP analysis was primarily focused on the CS and HK regions. The LF region, however, was analyzed based on sequencing.

The selected four combinations generated 12 polymorphic fragments and gave 33 length mutations primarily short length variants, presumably indels. For a single locus the number of alleles ranged from two to four. The CS–*Alw*26I combination proved to be the most polymorphic, with 12 distinct patterns.The other three combinations (CS–*Bsu*I, HK–*Rsa*I and HK–*Hinf*I) yielded 9, 7, and 5 different patterns, respectively (S4 Table).

Analysis of the polymorphism of plastid DNA markers (all four combinations together) revealed 99 different haplotypes across the 127 accessions studied. Nineteen haplotypes were shared by two or more accessions, while the remaining 80 were unique (private) haplotypes for the respective accessions. Among these, private haplotypes, 65 were found in Polish populations of *J. erucifolia*, and 15 were found in individuals of *J. erucifolia* subsp. *tenuifolia*. This high number of unique haplotypes reflects high intra-population variability.

Using all combinations of fragment-restriction enzyme for haplotype identification provides resolution that is too high to draw broader conclusions. Therefore, in the subsequent analysis, we focused on the CS-*Alw*26I combination, which generated 27 haplotypes (Table 5). Most populations were polymorphic and eight of them had unique haplotypes. Populations P (Pęczelice, Poland) as well as HT (Tápiószentmárton, Hungary) were monomorphic and represented by only one haplotype H1 and H5 respectively. The most common haplotype was H1, occurred in nine populations from Poland and individuals from Slovakia (20 individuals, frequency = 0.157), and was absent in other countries. Equally abundant was the H2 haplotype (6 populations, 12 individuals, frequency = 0.150), which occurred exclusively in Polish populations and was present regardless of subspecies affiliation. The third group of the most frequent haplotypes were haplotypes H3 and H19 (each present in 5 populations, in 11 and 12 individuals respectively). The four most frequent haplotypes (H1, H2, H3, and H19) were found in 62 individuals from various populations accounted for 48.8% of all individuals.

**Table 5:** Haplotype frequencies and composition of the populations of *Jacobaea erucifolia* (both subspecies) based on combination CS-*Alw*26I.

Haplotype	Population (number of individuals with haplotype)																					Total of indiv. with haplotype	Frequency
	Polish population of *J. erucifolia*										*J. erucifolia* subsp. *tenuifolia*												
	M	S	K	St	P	B	Sn	Brz	Si	Pa	HT	HI	HL	HP	NZ	SS	SB	SU	SL	SV	SZ		
H1	2	2	0	1	0	0	0	0	1	1	0	0	0	0	0	0	1	0	1	0	1	20	0.157
H2	4	0	3	0	0	3	3	0	4	2	0	0	0	0	0	0	0	0	0	0	0	19	0.150
H3	1	5	0	1	0	0	0	0	0	0	0	0	2	2	0	0	0	0	0	0	0	11	0.087
H4	2	0	0	0	0	0	0	0	0	3	0	0	0	0	0	0	0	0	0	0	0	5	0.039
H5	0	0	0	0	0	0	0	0	0	0	5	2	0	0	0	0	0	0	0	0	0	7	0.055
H6	0	0	3	0	0	0	0	2	0	0	0	0	0	0	0	0	0	0	0	1	0	6	0.047
H7	1	0	1	0	0	0	0	0	1	1	0	0	0	0	0	0	0	0	0	0	0	4	0.031
**H8**	0	0	0	0	0	0	**3**	0	0	0	0	0	0	0	0	0	0	0	0	0	0	3	0.024
H9	0	3	0	1	0	0	0	0	0	0	0	0	0	1	0	0	0	0	0	0	0	5	0.039
H10	0	0	1	0	0	0	2	0	1	0	0	0	0	0	0	0	0	0	0	0	0	4	0.031
**H11**	0	0	0	0	0	0	0	**3**	0	0	0	0	0	0	0	0	0	0	0	0	0	3	0.024
H12	0	0	0	0	0	0	0	3	0	0	0	0	0	0	0	0	0	1	0	0	0	4	0.031
H13	0	0	1	0	0	0	0	0	0	1	0	0	0	0	0	0	0	0	0	0	0	2	0.016
**H14**	0	0	0	0	0	0	0	0	0	0	0	0	0	0	2	0	0	0	0	0	0	2	0.016
H15	0	0	0	0	0	0	1	1	0	0	0	0	0	0	0	0	0	0	0	0	0	2	0.016
H16	0	0	0	0	0	0	0	0	0	0	0	1	1	0	0	0	0	0	0	0	0	2	0.016
**H17**	0	0	0	0	0	0	0	0	0	0	0	0	**1**	0	0	0	0	0	0	0	0	1	0.008
**H18**	0	0	0	0	0	0	0	0	0	0	0	0	0	**2**	0	0	0	0	0	0	0	2	0.016
H19	0	0	0	1	0	7	1	0	2	0	0	0	0	0	1	0	0	0	0	0	0	12	0.094
**H20**	0	0	0	**4**	0	0	0	0	0	0	0	0	0	0	0	0	0	0	0	0	0	4	0.031
H21	0	0	1	1	0	0	0	0	0	0	0	0	0	0	0	0	0	0	0	0	0	2	0.016
**H22**	0	0	0	0	0	0	0	0	0	0	0	0	**1**	0	0	0	0	0	0	0	0	1	0.008
H23	0	0	0	0	0	0	0	1	0	0	0	0	0	0	0	1	0	0	0	0	0	2	0.016
**H24**	0	0	0	0	0	0	0	0	**1**	0	0	0	0	0	0	0	0	0	0	0	0	1	0.008
**H25**	0	0	0	0	0	0	0	0	0	**1**	0	0	0	0	0	0	0	0	0	0	0	1	0.008
**H26**	0	0	0	0	0	0	0	0	0	**1**	0	0	0	0	0	0	0	0	0	0	0	1	0.008
**H27**	0	0	0	**1**	0	0	0	0	0	0	0	0	0	0	0	0	0	0	0	0	0	1	0.008
Total	10	10	10	10	10	10	10	10	10	10	5	3	5	5	3	1	1	1	1	1	1	127	1.000

Note: Population locations by acronyms are given in Table 1. Private haplotypes are highlighted in bold.

The remaining haplotypes were less common and unique to specific populations (11 haplotypes in total). Sixteen of the identified haplotypes were shared by two or more populations. The frequency of the private haplotypes ranged from 0.008 to 0.031 (Table 5). In the Sn, Brz, and St populations, private haplotypes were particularly abundant (specifically: H8, H11, and H20, respectively). The St and Pa populations exhibited the greatest haplotype richness, with as many as seven haplotypes identified in each. Moreover, the maximum number of private haplotypes (two) was also present in the St population and two other HL (Hungary) and Pa (Poland) populations. Reference individuals for the *tenuifolia* subspecies (SS, SB, SU, SL, SV, and SZ) shared haplotypes with Polish populations. In all populations representing the *tenuifolia* subspecies, the haplotype diversity was relatively low. Most populations in this group contained two haplotypes, with the exception of the HL population, where four haplotypes were found (two of which were unique). In Polish populations, haplotype diversity was much higher (5–7 in one population), and some haplotypes were not found in the populations of the *tenuifolia* subspecies. There was no haplotype common to all populations. Aside from the H2 haplotype, which occurred only in Polish populations (mostly), there was no clear pattern of haplotypes occurring exclusively in populations assigned to a given subspecies. When subspecies-specific haplotypes did occur, they were present in only 1–2 populations per group, and most were unique haplotypes (for details, see Table 5).

Detectable variation was also found in two other combinations: CS–*Bsu*RI, and HK–*Rsa*I + HK–*Hinf*I, (analyzed together). The analysis revealed the presence of 20 and 33 different haplotypes, respectively, with a significant number of them being unique (7 and 22, respectively). As before, most populations were polymorphic. Of the two monomorphic populations, both were represented by the H1 haplotype found in Hungarian populations. As previously noted, certain haplotypes appeared with higher frequency. In both combinations, the St population once again proved to be the most diverse. Information regarding haplotypes generated using the above combinations is provided in the supplementary materials (S1 and S2 Table).

**Fig 6:**
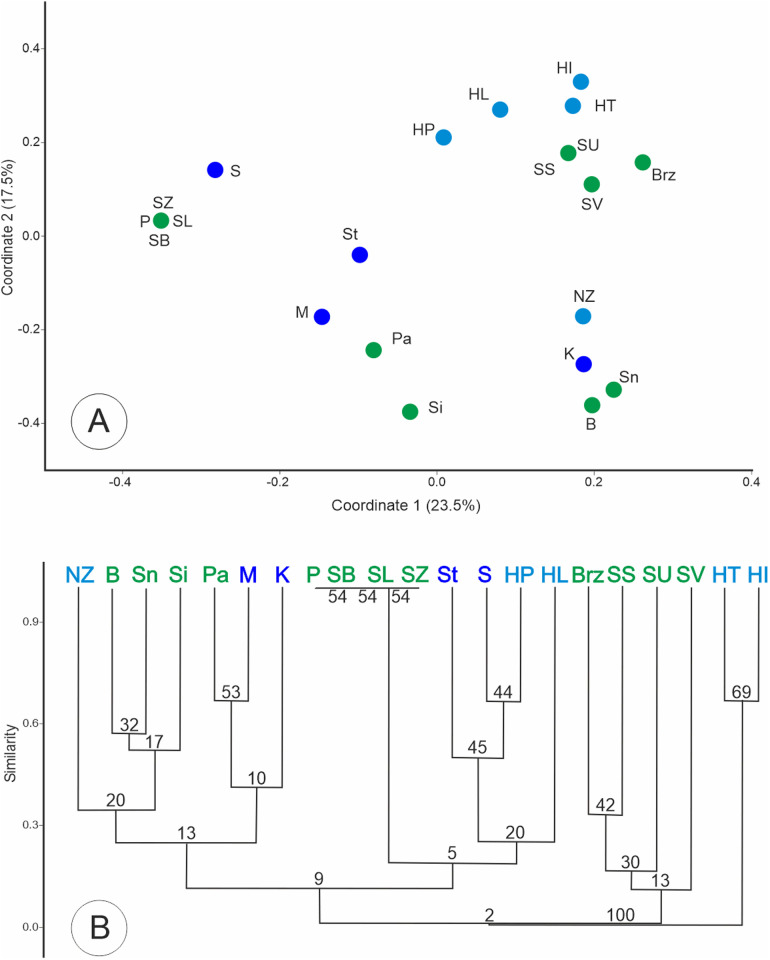
Grouping of populations of *J. erucifolia.*

Both the UPGMA dendrogram and PCoA analysis, based on a binary matrix of restriction fragment presence/absence, divided the populations into three groups. The smallest group consisted of two Hungarian populations (HT and HI). The two larger groups, further subdivided into smaller subgroups, included populations representing different subspecies. Notably, the reference group itself was split, with its members distributed across two distinct clusters (Fig 6).

Clustering based on principal component analysis of data on presence and proportions of chloroplast DNA haplotypes in populations (A) and UPGMA dendrogram (B). Names of populations refer to Table 1.

Individuals are represented by dots in colors, (red corresponds to *J. vulgaris*, blue to *J. erucifolia* subsp. *erucifolia*, green to *J. erucifolia* subsp. *tenuifolia* and sea blue for individuals/populations not assigned to a subspecies during collection, probably subsp. *tenuifolia*).

In summary, no taxonomic distribution of haplotypes was observed, dendrogram analysis did not reveal any relationship between the haplotypes present in a given population and its subspecies affiliation. The composition of the clusters formed suggests that the populations are more similar in terms of the habitats they occupy rather than their taxonomic classification (Table 1). Similarly, no clear geographical pattern of haplotypes distribution was observed in the study area.

### Analysis of chloroplast DNA and nuclear ribosomal DNA

The aligned sequences of the chloroplast intergenic spacer between *trnL* (UAA) exon and *trn*F (GAA) were about 960 bp in length. Sequences of 6 individuals constituting the reference group of *J. erucifolia* subsp. *tenuifolia*, and several individuals from both putative subspecies from Poland, Hungary, and Netherlands were compared. Moreover, individuals representing *J. vulgaris* from two Polish and one Dutch populations were included in the analysis. Notably, the latter group formed a cluster in the STRUCTURE analysis that included a significant proportion of alleles typical of *J. erucifolia.* Sequence comparisons revealed that the sequences of all analyzed *J. erucifolia* individuals, regardless of subspecies, were identical. Within the Dutch *J. vulgaris* group, most individuals exhibited sequences consistent with Polish *J. vulgaris* and those available in the GenBank database. However, two individuals (NP3 and NP7) identified as *J. vulgaris* displayed chromatogram additivity at sites with differential fixation between *J. vulgaris* and *J. erucifolia* (sequences comparison showed that they differed by four nucleotide substitutions and two 5-bp and 10-bp indels). For details check S3 Table.

To assess nuclear sequence-level variation, we also examined ITS sequences, which have been widely used in plant systematics and may help detect finer-scale divergence or historical gene flow. Finally, six ITS sequences from individuals representing the *tenuifolia* subspecies (reference group from Slovakia) were aligned and compared with sequences previously obtained [65] for *J. erucifolia* (both putative subspecies) from Poland. Sequence analysis of all individuals from the *J. erucifolia* group revealed no sequence differences.

## Discussion

Genetic diversity is the basis of the evolutionary process, and in the face of global climate change, it has become one of the most critical factors for monitoring the effects of climatic alterations on intraspecific genetic diversity. According to May [85], genetic variation is a fundamental component of biodiversity. Global climate change is widely expected to affect biodiversity, species distribution, and population growth in numerous ways. These effects include changes in population and species ranges [86], as well as impacts on phenotypic plasticity and adaptation in response to changing environmental conditions. Such changes may reduce genetic diversity within populations and species, thereby decreasing population viability and potentially leading to extinction [87,88]. Additionally, anthropogenic factors, such as land use changes, have a significant impact on migration patterns and adaptation to environmental conditions [87]. In light of this, more attention should be given to species whose populations are particularly vulnerable to these impacts, especially those at risk of extinction. Assessing the genetic diversity of species to determine the current patterns of population distribution and genetic variation is especially important considering the long-term consequences of current global environmental changes. Molecular methods, such as AFLP and cpDNA analyses, are invaluable tools for studying population structure and inter-population genetic diversity.

*Jacobaea erucifolia* is a rare species in Poland, occupying anthropogenically modified habitats such as roadsides, pastures, abandoned vineyards, and waste areas [17,89]. However, habitat disturbances can also create new ecological niches fostering the persistence of diverse forms, such as stabilized introgressants and homoploid hybrid species [89,90].

Two main subspecies (*erucifolia* and *tenuifolia*) have historically been recognized in Poland, primarily based on leaf shape and ecological preferences. *J. erucifolia* subsp. *tenuifolia* exhibiting a broader ecological range. The greatest difference between the two subspecies is the index of continentalism: *J. erucifolia* subsp. *erucifolia* is an Atlantic taxon, while the second subspecies is subcontinental. In terms of morphology, aside from leaf variability, the most notable differences (micromorphological) concern the hairs present on the surface of the achenes [91]. Although morphological and micromorphological analyses have shown the presence of both subspecies in Poland [21], no studies have yet demonstrated that these subspecies are genetically distinct. Furthermore, individuals classified as *tenuifolia* subspecies have not been compared with native populations occurring in neighboring regions.

RAPD-based analyses previously indicated that morphological subspecies in Poland are not genetically distinct. RAPD markers divided populations into two well-defined groups, with mixed clusters formed by individuals from both subspecies. Cluster analysis suggested that the grouping was influenced more by habitat similarity than genetic differentiation between the two taxa (S1 Fig). This justified the use of higher-resolution molecular markers such as AFLP and cpDNA, supported by additional sampling from other regions.

AFLP fingerprinting analysis revealed high polymorphism within populations and no consistent separation between subspecies. However, Polish populations (regardless of subspecies) exhibited a slightly higher degree of polymorphism compared to the others. Overall, the level of genetic diversity within the subspecies did not differ significantly, suggesting that their gene pools are not separate. Variation in sample sizes, especially in foreign populations, reflects differences in local population sizes and accessibility at the time of collection. While Polish populations were consistently represented by 10 individuals selected for broad geographic and ecological coverage, the number of individuals from Hungary, the Netherlands, and Slovakia was limited by available material. Although smaller sample sizes may influence some statistical outcomes, particularly estimates of diversity and structure, the consistency of clustering patterns across multiple methods supports the reliability of the overall genetic signal. Among the total amplified polymorphic products, none distinguished the two subspecies, and no unique products were classified as diagnostic or species-specific.

An AMOVA attributed most of the genetic variation to within populations (83–84%) rather than among populations or subspecies (15–16%), regardless of the type of analysis. The analysis indicated that most of the variation was due to differences between individuals within populations. This pattern aligns with previous findings for *J. vulgaris* and *J. aquatica* populations [17,92–94] where differences among individuals within populations also accounted for most of the variation. Further studies incorporating additional diversity metrics (e.g., Nei’s gene diversity, allelic richness) and confidence intervals could provide valuable information to better understand population-level variability and conservation potential. We consider this a promising direction for future research.

All analyses conducted using AFLP data (PCoA, UPGMA dendrogram, and NJ network) showed consistent results and divided all accessions into three main groups. One well-separated group consisted of *J. vulgaris* populations, which served as the out-group. The largest group encompassed individuals of *J. erucifolia* from Poland, forming a loose and mixed grouping without a clear subdivisions by subspecies or populations. The third group consisted of all foreign populations, which clustered together and notably included most individuals of the subsp. *tenuifolia* (reference group from Slovakia). Based on these results, we can assume that the populations from Hungary and the Netherlands (initially not assigned to a subspecies rank) also represent the *tenuifolia* subspecies.

Bayesian analyses using STRUCTURE grouped the accessions similarly to the previous analyses. Most Polish populations exhibited a high degree of admixture, with the exception of two populations (P and B) that stood out from the others and showed a low level of admixture. No significant differences were detected between populations tentatively classified as subspecies based on morphology, suggesting that their gene pools are not separate. Overall, population clustering revealed a consistent pattern: one group representing Polish *J. erucifolia* populations and another encompassing populations from the remaining regions. Moreover, unlike the foreign populations (from the Netherlands, Hungary, and Slovakia), most Polish *J. erucifolia* populations showed a noticeable overlap of gene pools. The STRUCTURE-defined clusters and admixture patterns suggest that Polish populations represent a genetically unified unit, despite morphological variation. This undermines the case for recognizing two subspecies.

The three-step STRUCTURE analysis was designed to address one of the main objectives of the study: clarifying the status of Polish populations, which until now had been assigned to subspecies solely on morphological grounds. Slovakian samples represented the only reference material with a confirmed taxonomic status (*J. erucifolia* subsp. *tenuifolia*). Therefore, analyzing Polish populations together with Slovakian material (Fig 4B) was a critical step for testing their genetic distinctiveness relative to a verified reference group. In contrast, material from Hungary and the Netherlands, although morphologically resembling subsp. *tenuifolia*, has not been formally assigned to subspecies and is referred to in our study only as *J. erucifolia*. For this reason, the intermediate analysis (Fig 4B) provided the necessary reference-based framework for interpreting the subsequent results when all foreign populations were included (Fig 4C).

Previous research [65] documented natural hybridization between *J. vulgaris* and *J. erucifolia* in Poland and the presence of hybrid populations. It also showed that introgression processes occur in both directions in the parental populations. The admixture of gene pools in both species is further confirmed by the current results. Both *J. erucifolia* populations show a slight admixture of the *J. vulgaris* gene pool, and several individuals from the *J. vulgaris* populations exhibited a slight admixture of the *J. erucifolia* gene pool. The most surprising result came from the Dutch population, which was morphologically classified as *J. vulgaris*. In previous analyses (PCoA, NJ), this population formed a subcluster (IB) related to the *J. vulgaris* group, but it occupied a position between this group and the *J. erucifolia* group formed by individuals from areas outside Poland. Half of the individuals in this population (classified as *J. vulgaris*) exhibited admixture, with both gene pools contributing to their genotypes, though the *J. erucifolia* cluster predominated. The remaining individuals were almost pure *J. erucifolia*. This might indicate that they are likely later-generation backcrosses containing a high proportion of *J. erucifolia*. In light of the above information, a more detailed and targeted analysis of hybridization—including the use of additional markers, quantification of introgression levels, and comparison of morphological traits between hybrids and non-hybrids would provide valuable insights. Moreover, such an integrated approach, combined with expanded geographic sampling, could significantly enhance our understanding of population dynamics and inform conservation strategies for *J. erucifolia* in Poland. Hybridization, especially under anthropogenic pressure, may play both adaptive and threatening roles for rare taxa. Our data are consistent with hybrid zones, asymmetrical gene flow, and potential for cryptic introgression.

All AFLP-based analyses mentioned above did not distinguish individuals belonging to particular subspecies within the Polish populations. Nevertheless, foreign populations assigned to subsp. *tenuifolia* have always formed a distinct, separate cluster. Therefore, this study challenges the status of subsp. *tenuifolia* in Poland.

According to some authors, the reliability of studies on complex genetic processes requires the collection of evidence from many sources [95–97]. For example, Boecklen and Howard [98] suggested that, in hybridization analyses, more than 70 diagnostic markers are needed to distinguish parent species from advanced backcrosses with sufficient certainty. On the other hand, highly backcrossed hybrids can be difficult to distinguish from their parents, as such individuals often cluster with pure individuals. Therefore, in addition to nuclear markers (AFLP), maternally inherited plastid markers were also analyzed using the PCR-RFLP method.

The highly conserved nature of the chloroplast genome is considered to be an ideal marker for population studies [99,100]. Although the rate of cpDNA evolution is slow, molecular analysis has shown that insertions, deletions, and point mutations cause detectable variation when using PCR-RFLP-based methods. In recent decades, plastid markers either species-specific or universal have been used to resolve phylogenetic relationships and species delimitations. The fact that they are maternally inherited makes them especially useful in research related to hybridization and introgression. They are also frequently used in analyses of representatives of the genus *Senecio* [101].

To characterize the haplotype diversity, its distribution in the studied populations and species, and to compare Poland with other parts of Europe, three fragments (CS, HK, and LF) were amplified using universal primer pairs developed by Demesure et al. [79] and Taberlet et al. [81], respectively. The resulting fragments were of the expected length, and no size differences were noted between the individuals in the studied populations. After digestion, the selected four fragment-enzyme combinations generated 12 polymorphic fragments, allowing the detection of 33 length mutations. The mutations detected in this study were mainly short-length variants, presumably indels. According to Clegg et al. [102]), the frequency of indels was higher than that of point mutations.

Analysis of the polymorphism of plastid DNA markers revealed 99 different haplotypes, the majority of which (80.8%) were unique. This result indicates significant diversity within individual populations, while also making it difficult to clearly interpret the similarities and differences between populations. Since each fragment-enzyme combination was sufficient to determine the haplotypes in the studied populations, the analysis focused on the most diverse combination, CS-*Alw*26I (supported by the results of the other combinations). Most populations were polymorphic, and in each fragment-enzyme combination, at least one haplotype was dominant and occurred at a higher frequency. These common haplotypes were found regardless of the region of origin of the individual populations or subspecies. However, no haplotype was common to all populations. Polish populations showed greater haplotype diversity compared to populations representing the *tenuifolia* subspecies. In conclusion, haplotypes did not exhibit a clear pattern of differentiation among the subspecies, and no clear regularity was observed in the geographic distribution of haplotypes.

In the case of the LF locus, the differences in the size of the fragments after digestion with restriction enzymes were so slight that it was uncertain whether they truly existed or were merely due to imperfections in the electrophoretic separation. To precisely determine the type of mutation within the theoretical variants, which may differ by only a few base pairs, sequencing was performed. Sequence comparison showed that the sequences of all analyzed individuals of *J. erucifolia* (from Poland, Slovakia, Hungary, and the Netherlands), regardless of subspecies, were identical. According to available data, chloroplast genomes of Asteraceae species do not show significant differences in size, gene content, or their arrangement [103]. However, this is largely not applicable to non-coding regions, where the differences can be significant. We demonstrated this in our previous publication [65], where sequence analysis of the LF fragment allowed the confirmation of hybrid presence in *Jacobaea* populations due to clear differences in the cpDNA sequences of the parent species. Furthermore, literature reports suggest that, for example, subspecies of *Senecio vulgaris* have different sequences of both cpDNA and rDNA [104]. In light of these data, the ITS sequences obtained in this study for reference individuals of the *tenuifolia* subspecies were also compared with those previously obtained for *J. erucifolia* (both subspecies; [65]). Again, no differences were found; the sequences were identical.

Finally, to resolve the ambiguous classification of individuals from population NP (identified as *J. vulgaris* in the field; however, STRUCTURE analysis suggested its affinity with *J. erucifolia*), LF fragments were sequenced and compared with those typical of both species *(J. vulgaris* and *J. erucifolia*). The sequences of some individuals were consistent with *J. vulgaris*, while two showed sequence differences typical of a hybrid origin. The AFLP results (see above) clearly indicated the probability that this population contained a mixture of *J. erucifolia*-like individuals, introgressed to different degrees.

The aim of our research was primarily to answer questions regarding both the condition of Polish populations of *J. erucifolia* in comparison with populations from other regions, as well as the taxonomic status of subspecies described solely based on morphological features.

Geographically and genetically distinct populations that exhibit clear phenotypic differences are assumed to be identified as subspecies [27,105] (see Introduction). Since intraspecific differentiation itself is often considered sufficient to designate a subspecies, there remains a significant open question regarding the patterns of variation that should be expected to confirm whether a given taxon truly merits subspecies rank.

In summary, detailed analysis of AFLP banding patterns allowed us to distinguish two distinct groups of individuals: one consisting of 10 Polish populations (both putative subspecies), and the other including the *tenuifolia* subspecies (Slovakia), as well as genetically and morphologically similar populations from Hungary and the Netherlands, currently also considered as the same subspecies. The Polish populations exhibited considerable intra-population diversity (a mixed group of both subspecies) and shared the same haplotypes. No diagnostic bands were found that would allow distinguishing the two subspecies within this group. Moreover, the lack of genetic divergence between these two subspecies was also confirmed by the results of studies based on nuclear and chloroplast DNA sequence analyses. In light of these results, we suggest that, at least in Poland, the subspecies status of *J. erucifolia* subsp. *tenuifolia* (genetically indistinct) is questionable.

The individuals constituting the second group of populations were clearly distinguished genetically from those from Poland. The populations from Hungary and the Netherlands, initially defined as *J. erucifolia* without indicating their subspecies affiliation, consistently grouped together with the subspecies *tenuifolia* from Slovakia. This grouping may confirm that they also represent the same subspecies. The fact that in all analyses based on AFLP markers, these populations were very clearly separated from the Polish representatives of *J. erucifolia*, proves their genetic distinctiveness. This, in addition to morphological differences, is one of the required conditions for recognizing them as a subspecies. Importantly, aside from a small number of fragments specific/unique to this subspecies (1.7%), no diagnostic products were found that would allow for distinguishing individuals forming both main groups (potential subspecies). Moreover, the sequencing results do not confirm the distinctness of these populations. When compared with the sequences obtained for representatives of the Polish populations, no differences were found in the cpDNA sequence, and in the case of individuals treated as reference, also in the rDNA sequence. So, the question arises: what are we dealing with?

Numerous studies have shown significant genetic differences, both at the sequence level and based on chloroplast and nuclear markers, between different subspecies [106–111]. According to Wang et al. [111], the degree of separation of the gene pools of subspecies may be related to the level of genetic diversity between them. The authors also observed that, although there was substantial genetic divergence among the subspecies studied, it varied from locus to locus across the genome. Furthermore, they found that the most information on subspecies delimitation came from a small part of the genome, while the rest was only slightly differentiated, indicating uneven gene flow and incomplete isolation. Since genetic distinctiveness has already been confirmed by AFLP analysis (foreign populations), the lack of clear differences within the analyzed sequences for subsp. *tenuifolia* does not necessarily undermine its taxonomic classification. It may, however, indicate the need for further analysis of other sequences.

Polish populations show significant phenotypic differentiation. Based on morphological features, two groups were distinguished, defined as two subspecies: *erucifolia* and *tenuifolia* (details above). However, analyses based on molecular markers did not support this. Although the main cluster formed by Polish populations was divided into smaller subclusters, both putative subspecies co-occurred in each of them, and the similarity of the habitats occupied seems to be the basis for their formation. The relationship with the environment was also indicated by earlier morphological studies. Examination of the geographic dependence of morphological differentiation in the taxa (using the same material) showed that population heterogeneity likely results from environmental factors [21]. Similarly, in molecular analyses, the Mantel test showed no significant relationship. Moreover, the comparison of Polish populations with populations representing the *tenuifolia* subspecies also excluded the occurrence of this subspecies in Poland. Therefore, the question arose: what could be the basis of this differentiation if it is not genetically determined? *J. erucifolia*, like other *Jacobaea* species, inhabits ruderal habitats, often altered by humans (urbanization), which certainly provokes adaptation processes that we can observe at various levels.

It is widely recognized that most organisms exhibit different phenotypes in response to different environmental factors. According to Sultan [46], each genotype has its own genetically determined level of phenotypic plasticity, with some traits being more conserved than others. Phenotypic plasticity, described as the ability of a single genotype to produce different phenotypes under variable environmental conditions, enables species persistence in novel or changing environments [46,112]. It is often linked to a mechanism that may facilitate invasions [47]. Unlike diagnostic traits for a given taxon, differences arising from phenotypic plasticity are not stable and disappear when plants are grown under the same conditions in common garden experiments [113]. In changing environments, phenotypic plasticity allows organisms to maintain their genotypes and continue to produce alternative phenotypes, in contrast to local adaptations that involve changes at the genetic level, creating long-lasting phenotypes and resulting in higher fitness. Local adaptation results in the formation of ecotypes, ecological races with distinct characteristics that provide them an environment-specific fitness advantage [114]. It typically requires changes at multiple loci (different alleles are favored in different environments), so the observed phenotypic differences between populations from different eco-regions are not accidental. They result from genetic differences arising from adaptation to the habitat [115]. Different ecotypes are often geographically close and interfertile, but there is no evidence of gene flow between populations of divergent ecotypes [116]. Ecotypes show strong differences in leaf morphology [117]. Some researchers perceive ecotypic differentiation as the first step towards the formation of new species [25,118,119]. However, we must remember that changes taking place in the environment not only result in speciation but also pose a threat to many species.

In our study, the populations of *J. erucifolia* from regions outside Poland (Slovakia, Hungary, and the Netherlands) are represented by the subspecies *tenuifolia*. Despite their clear genetic distinctiveness in relation to the Polish populations, some doubts arise from the fact that no diagnostic markers were found in their AFLP profiles. Such markers were also absent in the profiles of the Polish population group, which theoretically represents different subspecies. Therefore, it cannot be ruled out that we are dealing with the occurrence of ecotypes. Both groups seem to meet the criteria described above. Such varieties may occur within the same geographic region, where distinct habitats provide ecological niches. In the case of the Polish populations, the situation is more complex due to the internal morphological division. Therefore, we lean toward the interpretation that the morphological differences that led to the separation of the *tenuifolia* subspecies in Poland arose as a result of phenotypic plasticity. Both of these hypotheses require further research to confirm their validity.

In our analysis, we cannot overlook one of the most important causes of phenotypic differences: naturally occurring hybridization processes, which may go unnoticed or be incorrectly interpreted. This was pointed out by Abbott [48]. Naturally occurring hybrids are typically recorded based on morphology. There is a high probability that only the early generations of hybrids will be recognized as such, while later generations of backcrosses resembling one or both parents might be overlooked. Therefore, based solely on morphological analysis, hybrid zones that primarily include backcrosses and parental types will not be recognized in the wild. A good example of this is the Dutch population, whose individuals, designated as *J. vulgaris*, turned out to be a group of advanced backcrosses with a predominance of *J. erucifolia* admixture in our molecular studies. Furthermore, hybridization is believed to occur primarily between closely related, genetically similar species. Under normal circumstances, many of these species are isolated from one another by various barriers that prevent interbreeding and the formation of hybrid zones. However, natural or anthropogenic disturbances can disrupt these natural isolation mechanisms. According to literature data collected by Abbott [48], about one-third of hybrid zones occur in areas with disturbed environments.

Europe, like other continents, is influenced by global climate change, which, as we know, favors hybridization. Moreover, hybrids tend to adapt better to changing conditions. Among *Jacobaea* species, hybridization is common, and hybrids are not rare. Our previous research unequivocally confirmed the presence of hybrid populations between *J. erucifolia* and *J. vulgaris* [65], occurring in areas close to those currently studied. Polish populations are relatively small and scattered, and the species is considered endangered. Thus, the observation of mixed genotypes in almost all populations (as indicated by the STRUCTURE analysis) may raise concerns regarding biodiversity. Hybridization can threaten the existence of a rare species [120] by causing genetic assimilation of a rare taxon by a closely related and more common one. Arnold [121] defined this phenomenon as the loss of genotypes or phenotypes of a rare taxon due to asymmetric gene flow. It is worth emphasizing that our previous research showed a clear asymmetry toward *J. erucifolia* and a significant share of backcrosses in the group of analyzed hybrids. On the other hand, hybridization can prevent extinction by maintaining or increasing population size. This phenomenon is known as the “rescue effect” of hybridization [64,122] and is stronger when there is an asymmetry in the direction of introgression (backcrossing; [123]. Muhlfeld et al. [124] defined hybridization as an absorbing process that eventually leads to the point where all apparently phenotypically pure individuals (parental lines) may have hybrid origins, potentially resulting in the extinction of pure genomes. However, this process takes time and may remain unnoticed for an extended period. Moreover, according to O’Brien & Mayr [105], most subspecies are likely to be monophyletic; however, they may also arise from the hybridization of ancestral subspecies.

In the STRUCTURE analysis, the Polish populations formed a separate group. Apart from two populations (P and B), which were almost homogeneous, there was a clear overlap of two gene pools. One gene pool belonged to a quite homogeneous group corresponding to the subspecies *tenuifolia*. A slight admixture of the *J. vulgaris* gene pool was also present in most individuals from both groups comprising the *J. erucifolia* populations. It is known that genetically similar individuals tend to cluster together to form a species, subspecies, or population, but individuals can also form clusters if they are similarly mixed with other species.

To sum up, our research has shown that the morphological differences suggesting the presence of two subspecies in Poland are not determined by genetic differentiation and are likely the result of phenotypic plasticity and the *tenuifolia* subspecies does not occur in Poland. Our analyses revealed genetic differences (AFLP profiles) between Polish populations and those described as subspecies *tenuifolia* from neighboring countries. This differentiation, however, is not confirmed by the sequences analyzed (no differences). In combination with the lack of diagnostic markers, this is more consistent with their classification as ecotypes.

The presence of significant admixture in Polish *J. erucifolia* populations could be explained by alternative scenarios, such as, for instance, ancient hybridization events with other gene pools. If we assume that the traits considered diagnostic for both subspecies are largely the result of phenotypic plasticity, then we can question either their actual existence or the reliability of distinguishing them solely based on morphology. However, verification of any of these hypotheses requires further research, including a broader geographic sampling as well as the analysis of larger areas of the genome.

## Supporting information

S1 Fig UPGMA dendrogram of *Jacobaea erucifolia* (both putative subspecies) accessions based on RAPD molecular data. Cluster analysis based on the individual genetic distance calculated with 277 RAPD markers. (accessions codes as in Table 1).
(TIF)

S2 Fig ΔK plots for the STRUCTURE analyses of *Jacobaea erucifolia* (both subspecies) and *J. vulgaris* populations. (A) ten Polish populations of *J. erucifolia* with the reference group from Slovakia; (B) analysis including six additional populations of subsp. *tenuifolia* from Hungary and the Netherlands; (C) clustering analysis of all *J. erucifolia* and *J. vulgaris* populations examined. ΔK was calculated following the method of Evanno et al. [77].
(TIF)

S1 Table Haplotype frequencies and composition of the populations of *Jacobaea erucifolia* (both subspecies) based on combination CS-*Bsu*I. (DOCX)

S2 Table Haplotype frequencies and composition of the populations of *Jacobaea erucifolia* (both subspecies) based on the combined results of HK-*Rsa*I and HK-*Hinf*I combinations. (DOCX)

S3 Table Nucleotide positions in the aligned *trn*L-F intergenic spacer sequences that differ between *Jacobaea vulgaris*, *Jacobaea erucifolia*, and their putative hybrids (NP3, NP7). (DOCX)

S4 Table Major patterns and variants (in bp) of fragments revealed in each polymorphic site detected using different primer pair-restriction enzyme combinations in accessions of J. erucifolia studied. (DOCX)
