# Supplementary figures and images for: Morphological differences in populations of Jacobaea erucifolia: Genetic differentiation, phenotypic plasticity or ecotypes?
Source: PLoS One. 2025 Sep 23;20(9):e0332808. doi: 10.1371/journal.pone.0332808 (PMC12456790; doi:10.1371/journal.pone.0332808)

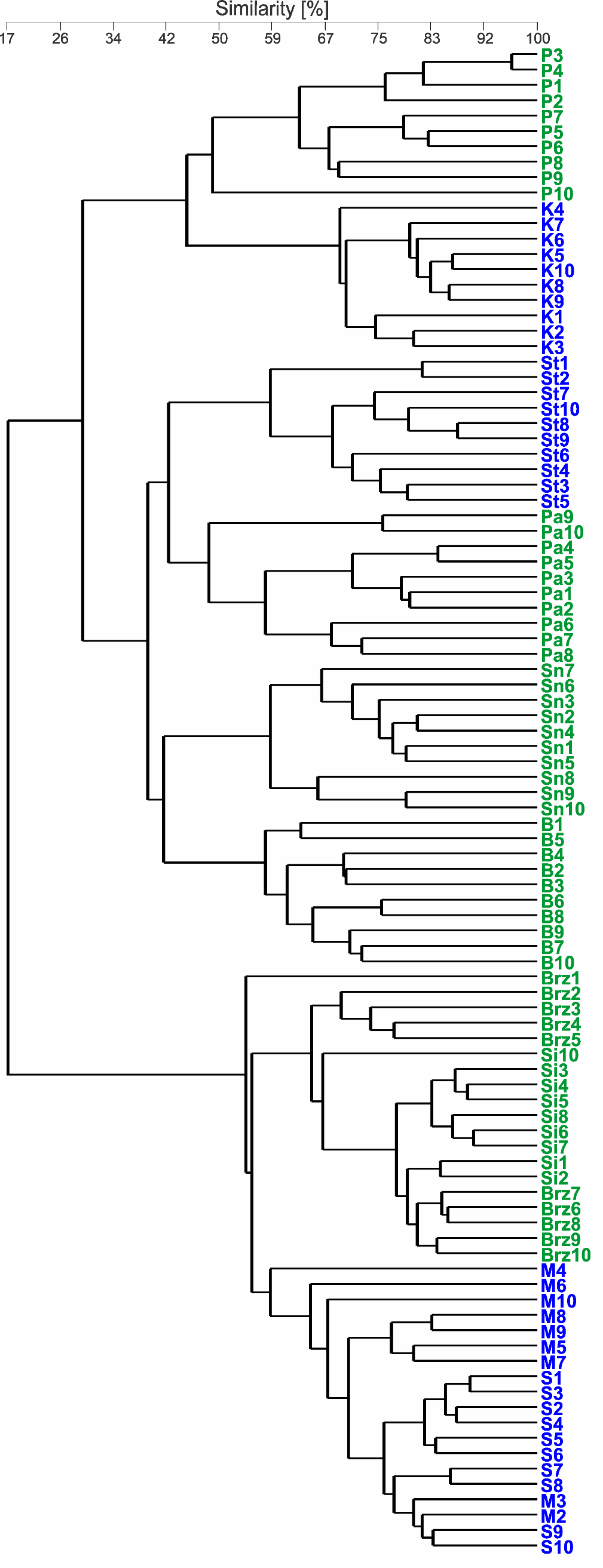

Supplement: S1 Fig — Cluster analysis based on the individual genetic distance calculated with 277 RAPD markers. (accessions codes as in Table 1). (TIF) [file pone.0332808.s001.tif]

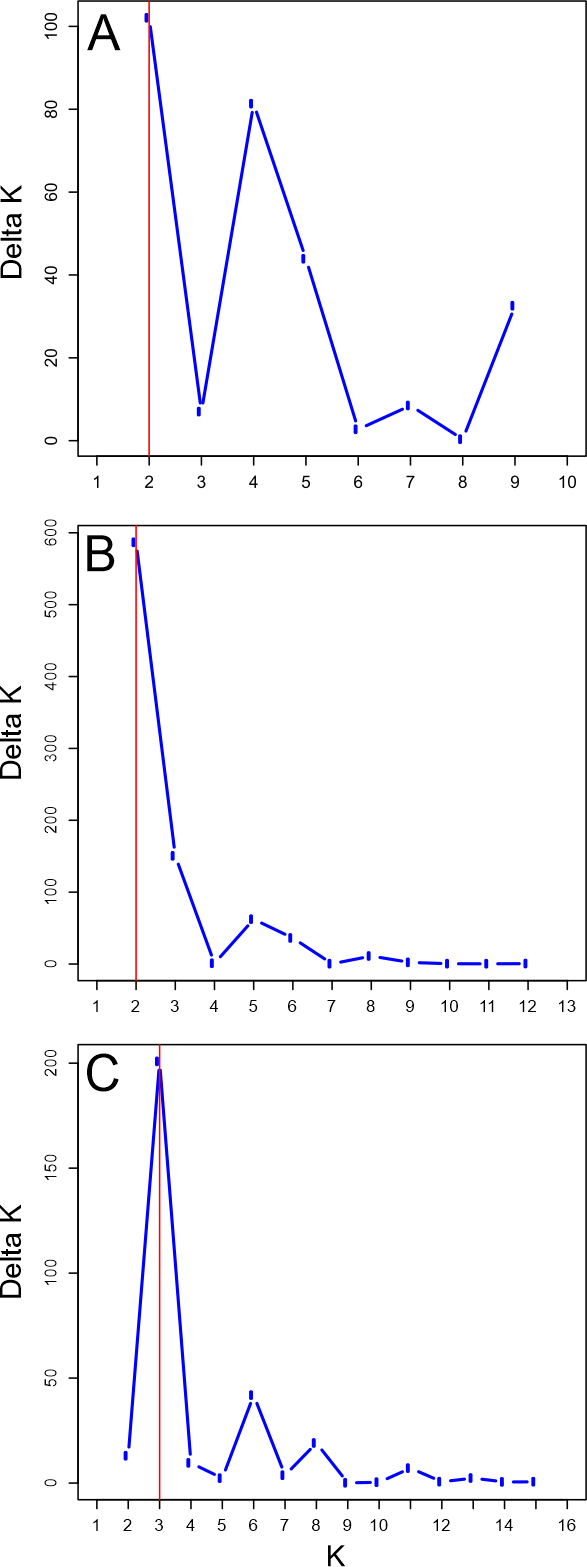

Supplement: S2 Fig — (A) ten Polish populations of J. erucifolia with the reference group from Slovakia; (B) analysis including six additional populations of subsp. tenuifolia from Hungary and the Netherlands; (C) clustering analysis of all J. erucifolia and J. vulgaris populations examined. ΔK was calculated following the method of Evanno et al. [77]. (TIF) [file pone.0332808.s002.tif]
