# Supplementary material for: Morphological differences in populations of Jacobaea erucifolia: Genetic differentiation, phenotypic plasticity or ecotypes?
Source: PLoS One. 2025 Sep 23;20(9):e0332808. doi: 10.1371/journal.pone.0332808 (PMC12456790; doi:10.1371/journal.pone.0332808)
Supplement: S1 Table — (DOCX) [file pone.0332808.s003.docx]

S1 Table Haplotype frequencies and composition of the populations of *Jacobaea erucifolia* (both subspecies) based on combination CS-*Bsu*I

| **Haplotype** | **Population (number of individuals with haplotype)** | | | | | | | | | | | | | | | | | | | | | **Total of indiv. with haplotype** | **Frequency** |
| --- | --- | --- | --- | --- | --- | --- | --- | --- | --- | --- | --- | --- | --- | --- | --- | --- | --- | --- | --- | --- | --- | --- | --- |
|  | **Polish population of *J. erucifolia*** | | | | | | | | | | ***J. erucifolia* subsp. *tenuifolia*** | | | | | | | | | | |  |  |
|  | **M** | **S** | **K** | **St** | **P** | **B** | **Sn** | **Brz** | **Si** | **Pa** | **HT** | **HI** | **HL** | **HP** | **NZ** | **SS** | **SB** | **SU** | **SL** | **SV** | **SZ** |  |  |
| H1 | 4 | 0 | 0 | 0 | 0 | 0 | 5 | 4 | 9 | 9 | 5 | 3 | 3 | 4 | 0 | 0 | 0 | 0 | 0 | 0 | 0 | 46 | 0.362 |
| H2 | 0 | 8 | 0 | 0 | 0 | 0 | 3 | 1 | 0 | 1 | 0 | 0 | 1 | 0 | 0 | 0 | 0 | 0 | 0 | 0 | 0 | 14 | 0.110 |
| H3 | 6 | 2 | 0 | 1 | 0 | 0 | 0 | 0 | 0 | 0 | 0 | 0 | 0 | 0 | 0 | 0 | 0 | 0 | 0 | 0 | 0 | 9 | 0.071 |
| **H4** | 0 | 0 | **7** | 0 | 0 | 0 | 0 | 0 | 0 | 0 | 0 | 0 | 0 | 0 | 0 | 0 | 0 | 0 | 0 | 0 | 0 | 7 | 0.055 |
| H5 | 0 | 0 | 0 | 3 | 0 | 3 | 0 | 1 | 0 | 0 | 0 | 0 | 0 | 0 | 0 | 0 | 0 | 0 | 0 | 0 | 0 | 7 | 0.055 |
| H6 | 0 | 0 | 0 | 0 | 0 | 3 | 2 | 0 | 0 | 0 | 0 | 0 | 0 | 0 | 0 | 0 | 0 | 0 | 0 | 0 | 0 | 5 | 0.039 |
| H7 | 0 | 0 | 0 | 0 | 0 | 0 | 0 | 0 | 0 | 0 | 0 | 0 | 0 | 0 | 3 | 0 | 0 | 1 | 0 | 0 | 0 | 4 | 0.031 |
| **H8** | 0 | 0 | 0 | 0 | **5** | 0 | 0 | 0 | 0 | 0 | 0 | 0 | 0 | 0 | 0 | 0 | 0 | 0 | 0 | 0 | 0 | 5 | 0.039 |
| H9 | 0 | 0 | 1 | 0 | 0 | 0 | 0 | 0 | 0 | 0 | 0 | 0 | 1 | 1 | 0 | 1 | 0 | 0 | 0 | 0 | 0 | 4 | 0.031 |
| **H10** | 0 | 0 | 0 | 0 | 0 | 0 | 0 | 0 | 0 | 0 | 0 | 0 | 0 | 0 | 0 | 0 | 1 | 0 | 1 | 0 | 1 | 3 | 0.024 |
| H11 | 0 | 0 | 0 | 2 | 0 | 0 | 0 | 0 | 1 | 0 | 0 | 0 | 0 | 0 | 0 | 0 | 0 | 0 | 0 | 0 | 0 | 3 | 0.024 |
| **H12** | 0 | 0 | 0 | 0 | 0 | 0 | 0 | **3** | 0 | 0 | 0 | 0 | 0 | 0 | 0 | 0 | 0 | 0 | 0 | 0 | 0 | 3 | 0.024 |
| H13 | 0 | 0 | 0 | 1 | 3 | 0 | 0 | 0 | 0 | 0 | 0 | 0 | 0 | 0 | 0 | 0 | 0 | 0 | 0 | 0 | 0 | 4 | 0.031 |
| H14 | 0 | 0 | 0 | 0 | 0 | 2 | 0 | 0 | 0 | 0 | 0 | 0 | 0 | 0 | 0 | 0 | 0 | 0 | 0 | 1 | 0 | 3 | 0.024 |
| **H15** | 0 | 0 | 0 | **2** | 0 | 0 | 0 | 0 | 0 | 0 | 0 | 0 | 0 | 0 | 0 | 0 | 0 | 0 | 0 | 0 | 0 | 2 | 0.016 |
| H16 | 0 | 0 | 0 | 1 | 1 | 0 | 0 | 0 | 0 | 0 | 0 | 0 | 0 | 0 | 0 | 0 | 0 | 0 | 0 | 0 | 0 | 2 | 0.016 |
| H17 | 0 | 0 | 1 | 0 | 0 | 1 | 0 | 0 | 0 | 0 | 0 | 0 | 0 | 0 | 0 | 0 | 0 | 0 | 0 | 0 | 0 | 2 | 0.016 |
| H18 | 0 | 0 | 0 | 0 | 0 | 1 | 0 | 1 | 0 | 0 | 0 | 0 | 0 | 0 | 0 | 0 | 0 | 0 | 0 | 0 | 0 | 2 | 0.016 |
| **H19** | 0 | 0 | 0 | 0 | **1** | 0 | 0 | 0 | 0 | 0 | 0 | 0 | 0 | 0 | 0 | 0 | 0 | 0 | 0 | 0 | 0 | 1 | 0.008 |
| **H20** | 0 | 0 | **1** | 0 | 0 | 0 | 0 | 0 | 0 | 0 | 0 | 0 | 0 | 0 | 0 | 0 | 0 | 0 | 0 | 0 | 0 | 1 | 0.008 |
| Total | 10 | 10 | 10 | 10 | 10 | 10 | 10 | 10 | 10 | 10 | 5 | 3 | 5 | 5 | 3 | 1 | 1 | 1 | 1 | 1 | 1 | 127 | 1.000 |

Note: Population locations by acronyms are given in Table 1. Private haplotypes are highlighted in bold.
