# Supplementary material for: Morphological differences in populations of Jacobaea erucifolia: Genetic differentiation, phenotypic plasticity or ecotypes?
Source: PLoS One. 2025 Sep 23;20(9):e0332808. doi: 10.1371/journal.pone.0332808 (PMC12456790; doi:10.1371/journal.pone.0332808)
Supplement: S3 Table — (DOCX) [file pone.0332808.s005.docx]

S3 Table Nucleotide positions in the aligned *trn*L-F intergenic spacer sequences that differ between *Jacobaea vulgaris*, *Jacobaea erucifolia*, and their putative hybrids (NP3, NP7).

|  | **Variable sites** | | | | | |
| --- | --- | --- | --- | --- | --- | --- |
|  | **280** | **317-321** | **514** | **588** | **699** | **769-778** |
| *J. vulgaris* G7 | G | - - - - - | C | C | G | GCATGAGACT |
| *J. vulgaris* NP3, NP7 | T | - - - - - | C | C | C | - - - - - - - - - - |
| *J. erucifolia* SB | T | AGAAA | T | A | C | - - - - - - - - - - |
