# Supplementary material for: Morphological differences in populations of Jacobaea erucifolia: Genetic differentiation, phenotypic plasticity or ecotypes?
Source: PLoS One. 2025 Sep 23;20(9):e0332808. doi: 10.1371/journal.pone.0332808 (PMC12456790; doi:10.1371/journal.pone.0332808)
Supplement: S4 Table — (DOCX) [file pone.0332808.s006.docx]

S4 Table Major patterns and variants (in bp) of fragments revealed in each polymorphic site detected using different primer pair-restriction enzyme combinations in accessions of *J. erucifolia* studied.

| Polymorphic  sites | Length  major pattern/variant (bp) |
| --- | --- |
| CS – (*Alw*26) 1 | 590 (C)/595 (B), 580 (D), 600 (A) |
| CS – (*Alw*26) 2 | 370 (A)/360 (B) |
| CS – (*Alw*26) 3 | 345 (A)/335 (B) |
| CS – (*Alw*26) 4 | 280 (B)/290 (A), 270 (D), 275 (C) |
| CS – (*Bsu* I) 1 | 685 (C)/735 (B), 800 (A) |
| CS – (*Bsu* I) 2 | 560 (B)/580 (A) |
| CS – (*Bsu* I) 3 | 285 (D)/290 (C), 325 (A), 320 (B) |
| HK – (*Rsa* I) 1 | 920 (B)/885 (C), 940 (A) |
| HK – (*Rsa* I) 2 | 680 (A)/670 (B) |
| HK – (*Rsa* I) 3 | 260 (A)/250 (B) |
| HK – (*Hinf* I) 1 | 715 (B)/700 (C), 745 (A) |
| HK – (*Hinf* I) 2 | 240 (A)/230 (B) |

The different polymorphisms in each polymorphic site are designated by the letters A, B, C, and D
